# Rapid α-oligomer formation mediated by the Aβ C terminus initiates an amyloid assembly pathway

**DOI:** 10.1038/ncomms12419

**Published:** 2016-08-22

**Authors:** Pinaki Misra, Ravindra Kodali, Saketh Chemuru, Karunakar Kar, Ronald Wetzel

**Affiliations:** 1 Structural Biology Department, University of Pittsburgh School of Medicine, Pittsburgh, Pennsylvania 15260, USA; 2 Pittsburgh Institute for Neurodegenerative Diseases, University of Pittsburgh School of Medicine, Pittsburgh 15260, Pennsylvania, USA

## Abstract

Since early oligomeric intermediates in amyloid assembly are often transient and difficult to distinguish, characterize and quantify, the mechanistic basis of the initiation of spontaneous amyloid growth is often opaque. We describe here an approach to the analysis of the Aβ aggregation mechanism that uses Aβ-polyglutamine hybrid peptides designed to retard amyloid maturation and an adjusted thioflavin intensity scale that reveals structural features of aggregation intermediates. The results support an aggregation initiation mechanism for Aβ-polyQ hybrids, and by extension for full-length Aβ peptides, in which a modular Aβ C-terminal segment mediates rapid, non-nucleated formation of α-helical oligomers. The resulting high local concentration of tethered amyloidogenic segments within these α-oligomers facilitates transition to a β-oligomer population that, via further remodelling and/or elongation steps, ultimately generates mature amyloid. Consistent with this mechanism, an engineered Aβ C-terminal fragment delays aggregation onset by Aβ-polyglutamine peptides and redirects assembly of Aβ_42_ fibrils.

## Abstract (web summary)

The elucidation of amyloid nucleation mechanisms remains challenging as early oligomeric intermediates are transient and difficult to distinguish. Here the authors use Aβ- polyglutamine hybrid peptides designed to slow and limit amyloid maturation to provide insights into the structures of Aβ self-assembly intermediates.

## Graphical abstract

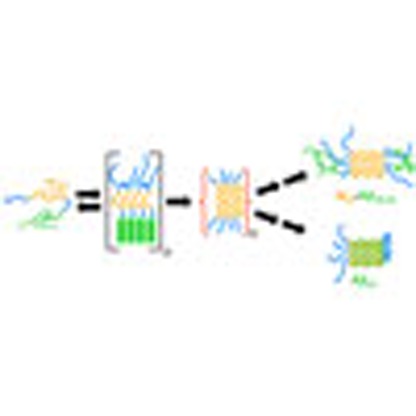

In Alzheimer's disease and other amyloid-associated conditions^1^, it is critically important to understand the mechanisms by which amyloid formation is initiated and the extent to which intermediate oligomeric species contribute to amyloid formation and cytotoxicity. Elucidation of amyloid nucleation mechanisms is especially challenging, however, in systems that feature oligomeric intermediates^2^,^3^,^4^ and secondary nucleation^5^ pathways. For different proteins, nucleation of amyloid formation might proceed either within an on-pathway oligomeric intermediate^6^ or via a classical nucleated growth polymerization^5^ featuring the direct formation of rare amyloid-like conformations in monomers^7^,^8^,^9^,^10^ or small multimers^8^.

Most mechanisms proposed to account for Aβ amyloid nucleation invoke an on-pathway role for one or more oligomeric assembly intermediates, but the structural details of these transformations remain mysterious. One early proposal was that amyloid nucleation is mediated by self-association of curvilinear protofibrillar intermediates^3^. Alternatively, observation of spherical oligomeric intermediates preceding Aβ protofibril and fibril formation^2^,^11^ suggested that spontaneous Aβ amyloid formation might proceed via a nucleated conformational conversion mechanism in which oligomer rearrangements serve both as the source of amyloid nucleation and as a means of fibril elongation^12^,^13^. Other mechanisms have been elucidated for the role of oligomers in formation of other amyloid fibrils^6^. Aβ oligomerization begins from intrinsically disordered monomers^14^, which progress through sub-populations of metastable multimers^15^ and transient oligomers exhibiting high α-helix contents^16^ and low ThT responses^13^,^17^,^18^ consistent with low amyloid-like β-structure. Based in part on earlier reports of transient formation of α-oligomers during Aβ fibril growth^16^, a general mechanism has been proposed for initiation of amyloid assembly (Fig. 1a) in some peptides in which early formation of α-helical oligomers leads to a high local concentration of an adjacent disordered segment, overcoming the concentration barrier to amyloid nucleation^19^. Once amyloid begins to grow, the α-helical segment appears to quickly unravel to join in the β-sheet network of the mature fibrils^20^,^21^ (Fig. 1a). This rapid annealing makes it very challenging to obtain direct structural evidence to support a role for early α-helical intermediates.

Intriguingly, an almost identical mechanism was deduced for the nucleation of polyglutamine (polyQ) amyloid formation in the Huntingtin (HTT) exon1-like fragments implicated in Huntington's disease^22^. In this mechanism (Fig. 1b), the 17 amino acid HTT^NT^ segment of HTT exon1 readily undergoes a polyQ repeat length-dependent transition from disordered monomer to α-helix rich tetramer and higher oligomers^22^,^23^,^24^. In these non-β aggregates, the HTT^NT^ segments act as quasi-independent, modular units to form α-helical bundles while the tethered, largely disordered polyQs are brought together within the oligomers at a high local concentration that greatly facilitates polyQ amyloid nucleation. Evidence in support of this mechanism includes (a) a dramatic rate increase on polyQ amyloid formation by covalent attachment of HTT^NT^, (b) early formation of ThT-negative oligomeric intermediates and (c) a unique, very low concentration dependence of initial aggregation rates that is inconsistent with a classical nucleated growth polymerization mechanism^22^. The rate enhancement by HTT^NT^ has a modular aspect in that can be observed whether it is attached to the N terminus or C terminus of a polyQ track, and whether or not there is an insertion of Lys residues between the HTT^NT^ and the polyQ^22^. With or without attached polyQ, HTT^NT^ readily forms tetramers and metastable, α-helix rich oligomers^23^,^24^. Amyloid fibrils of HTT exon1-like peptides have a polyQ core but retain some HTT^NT^-associated α-helical elements^25^,^26^ whose interactions provide additional stability to the fibrils^27^. Finally, introduction of isolated HTT^NT^ peptides in *trans* delays onset of aggregation, presumably via formation of mixed oligomers^28^ (Fig. 1d). In contrast to the experimental challenges to demonstrating the Aβ amyloid nucleation mechanism (Fig. 1a), elucidation of the analogous mechanism for HTT exon1 (Fig. 1b) was greatly aided by several features, including a low tendency of HTT^NT^ itself to make amyloid^29^ and therefore to remain in stable α-helical oligomers^23^, the relatively slow aggregation kinetics of isolated polyQ sequences, and the strong tendency of polyQ amyloid to exclude flanking sequences from the developing amyloid core^25^,^26^.

In this paper, we take advantage of these features of polyQ to test the Fig. 1a hypothesis that a segment of Aβ might act similarly to HTT^NT^ as a modular unit to mediate formation of non-β oligomeric intermediates required for efficient amyloid nucleation by an adjacent sequence (Fig. 1a). Our results provide broad support for this hypothesis while suggesting a mechanistic rationale for the dramatic difference in amyloidogenicity between Aβ_42_ and Aβ_40_. In addition, an appreciation of the strong dependence of ThT intensity on amyloid-like β-structure content reveals some surprising features of the assembly mechanism.

## Results

### Assigning pro-α and pro-β segments within the Aβ molecule

**Figure 1:**
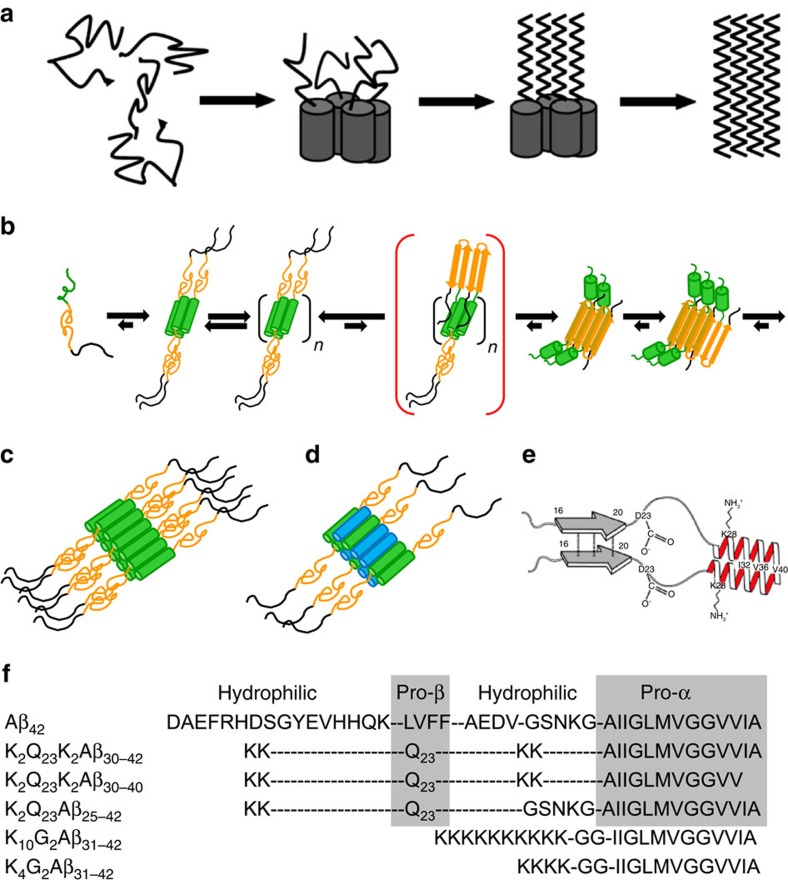
Model mechanisms and peptides. (**a**) Generic mechanistic model for α-helical oligomer mediated amyloid nucleation (reprinted with permission from Oxford University Press; ref. 19. (**b**) Proposed mechanism for α-helical oligomer mediated amyloid nucleation of HTT exon1-like fragments (reprinted from ref. 24), with green=HTT^NT^; orange=polyQ; black=proline rich domain. (**c**) Model for an HTT exon1 homo-oligomer (reprinted with permission from Elsevier Ltd; ref. 28^28^). (**d**) Model for hetero-oligomer generated from a mixture of HTT exon1 and HTT^NT^ (blue) (reprinted with permission from Elsevier Ltd; ref. 28^28^). (**e**) Structural model for Aβ in inclusion bodies from bacterial expression based on FTIR, solid-state NMR and hydrogen/deuterium exchange solution NMR (reprinted with permission from WILEY-VCH Verlag GmbH & Co.; ref. 43^43^). (**f**) Model peptides used in this study. Note that **a**–**d** are purely schematic and are not intended to imply particular knowledge of either the multimer sizes or the parallel or anti-parallel arrangement of the helices.

We first had to define the most likely segments of Aβ to serve the various roles suggested by the generic mechanism in Fig. 1a, including (a) the pro-α segment responsible for rapid, initial assembly into a helical, oligomeric intermediate and (b) the pro-β segment responsible for rapid nucleation of amyloid structure within that intermediate. Solution NMR of the monomer in aqueous solution provides no clues, revealing a completely disordered peptide^14^. The relatively hydrophilic nature and experimental accessibility of the Aβ N terminus (Fig. 1f) in various assembled states^30^ suggests that this segment is unlikely to play a central role in the assembly mechanism. In contrast, NMR studies of monomeric Aβ in the presence of SDS^31^ and lyophilized Aβ oligomers^32^ show that both the 10–24 and 28–42 regions of Aβ are capable of forming stable α-helix, thus suggesting that either might be capable of serving as the pro-α segment. At the same time, a variety of studies suggest that the most amyloid-prone portion of Aβ is the 16–20 Leu-Val-Phe-Phe (LVFF) segment^33^,^34^,^35^. It therefore seemed most likely that the central portion of Aβ including LVFF is the segment that initiates amyloid structure formation within oligomers, while the C-terminal Aβ segment mediates initial α-helical oligomer formation (Fig. 1f).

Based on these assignments we designed three hybrid peptides (Fig. 1f) to test the Fig. 1a mechanism. To replace the presumptive Aβ pro-β segment, we chose a polyQ repeat length of Q_23_, since a simple K_2_Q_23_K_2_ peptide aggregates very slowly^8^. We maintained the Aβ C-terminal segments at the C termini of the designed hybrid peptides, to give the critical difference between Aβ_40_ and Aβ_42_ the greatest opportunity to express itself at the peptide C termini, in analogy to the Aβ peptides. A Lys-Lys pair was inserted N-terminal to the polyQ, to replace the hydrophilic Aβ N-terminal domain. In addition, to mimic the hydrophilic Aβ_21–28_ segment and thereby further improve peptide kinetic solubility we inserted a Lys–Lys sequence between the polyQ and Aβ portions in two of the peptides. We justify the latter insertion based on our model (Fig. 1b) that pro-α segments act in a modular way in the nucleation mechanism, and on previous data that helped generate that model which show that a Lys–Lys insert between HTT^NT^ and polyQ has little effect on the ability of HTT^NT^ to enhance aggregation kinetics^22^. Nonetheless, to ensure that the Lys–Lys insert is not playing an outsized role in the hybrid Aβ-polyQ peptides, we also designed a third peptide in which we left out the Lys–Lys insert and replaced it with the wild-type Aβ GSNK segment. This design had the added benefit of testing a possible mechanistic role of GSNK, which has been suggested to influence Aβ monomer conformation^36^ and amyloid assembly kinetics and structure^20^,^35^,^37^.

This led to a total of three designed peptides, each harbouring presumptive pro-α segments derived from Aβ and an installed polyQ pro-β segment meant to replace the presumptive pro-β LVFF segment of Aβ (Fig. 1).

### Behaviour of K_2_Q_23_K_2_Aβ_30–42_

**Figure 2:**
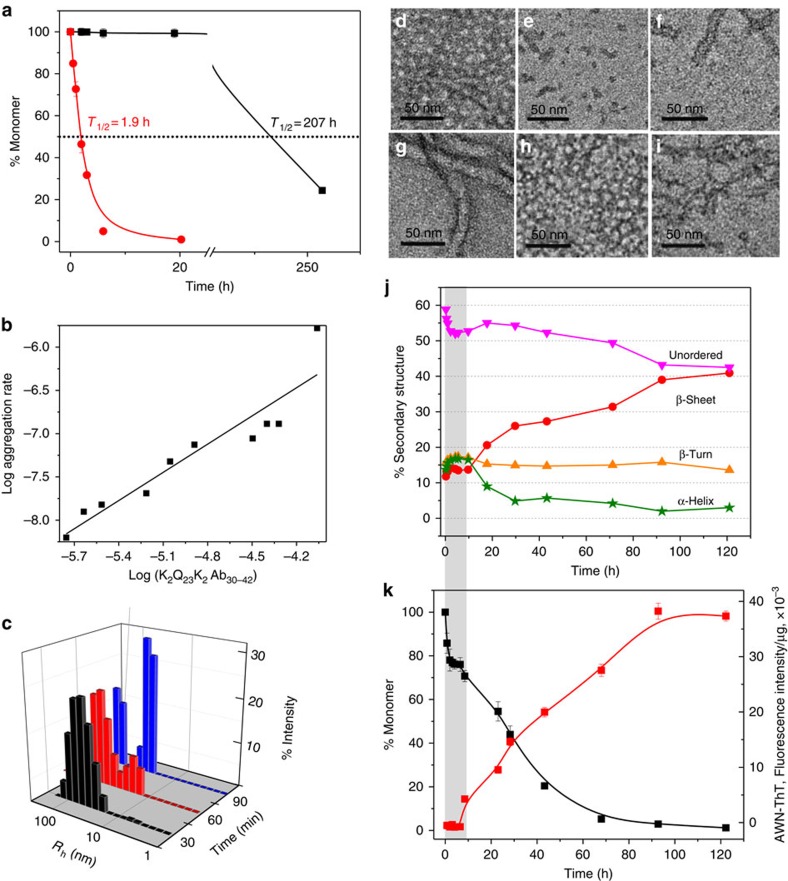
Aggregation of K_2_Q_23_K_2_Aβ_30–42_. (**a**) Aggregation assessed by disappearance of monomer for 37 °C incubation in PBS of 87 μM K_2_Q_23_K_2_Aβ_30–42_ (red circle) compared with 103 μM K_2_Q_23_K_2_ (black square). (**b**) Log–log plot of initial aggregation rates of K_2_Q_23_K_2_Aβ_30–42_ at various starting concentrations (Table 1 and Supplementary Fig. 1a). (**c**) Particle histograms from DLS analysis at different incubation times of K_2_Q_23_K_2_Aβ_30–42_ reaction described in **a** (Supplementary Fig. 2a). (**d**–**i**) TEM images (scale bar, 50 nm) of incubation of K_2_Q_23_K_2_Aβ_30–42_ at 37 °C in PBS at either 87 μM (**d**, 30 min; **e**, 90 min; **f**, 180 min; **g**, 2,880 min) or 6 μM (**h**, 2 h; **i**, 5 h). (**j**) Time-dependent changes in secondary structure content from CD analysis (Supplementary Fig. 3a) of reaction mixtures of the incubation of 6 μM K_2_Q_23_K_2_Aβ_30–42_ at 37 °C in PBS (120 h, anti-parallel β-sheet=41%, parallel β-sheet=0%); (**k**) aggregation kinetics of the reaction described in **j** as assessed by changes in monomer concentration (black square) and weight-normalized ThT fluorescence (AWN-ThT) (red circle). The grey-shaded bar spanning **j** and **k** highlights the time domain in which the α-helix content increases while ThT fluorescence is nil.

Addition of the Aβ_30–42_ fragment to K_2_Q_23_K_2_ markedly increases its spontaneous aggregation, in a manner similar to the effect of HTT^NT^ on polyQ sequences^22^. Thus, while 103 μM K_2_Q_23_K_2_ exhibits a long lag time before onset of aggregation with a *t*_½_ of aggregation of 207 h (Fig. 2a, black square), a similar concentration of K_2_Q_23_K_2_Aβ_30–42_ aggregates with a *t*_½_ of 1.9 h and no lag time (Fig. 2a, red circle). The concentration dependence of initial aggregation rates (Supplementary Fig. 1) in a log–log plot (a way to dissect some nucleation mechanisms^5^) gave a slope of 1.1 (Fig. 2b; Table 1) for this peptide. This is in the same range as the signature shallow slope exhibited by HTT^NT^-polyQ peptides^22^ and contrasts with the slope of 5.9 (Table 1) for K_2_Q_23_K_2_ itself that translates to a critical nucleus (n*) of 4 (ref. 8). In further contrast with K_2_Q_23_K_2_, which exhibits no detectible aggregates by DLS or EM during the lag phase^8^, K_2_Q_23_K_2_Aβ_30–42_ at very early time points generates aggregates with the size, shape and staining characteristics of spherical oligomers and protofibrils as described for Aβ_42_ (ref. 18). Thus, in a time frame where about 15% of the reaction mixture can be sedimented by centrifugation (Fig. 2a, red circle), we observed relatively small particles by EM (spherical oligomers of 10–20 nm; Fig. 2d) and DLS (∼60 nm; Fig. 2c and Supplementary Fig. 2).

To more closely dissect the reaction time course of K_2_Q_23_K_2_Aβ_30–42_, we slowed the kinetics using a 6 μM starting concentration. In this and other assembly reactions reported here, we exclusively used our adjusted measure of ThT fluorescence, an ‘aggregate-weight-normalized (AWN)' ThT intensity, which is the raw ThT fluorescence units divided by the mole fraction of starting monomer that is in pelletable aggregates at each time point^18^,^38^ (Methods). We found that K_2_Q_23_K_2_Aβ_30–42_ undergoes a rapid drop in monomer concentration within the first 2 h of incubation, after which there is little further change in monomer concentration up to 10 h (Fig. 2k, black square). Interestingly, aggregates formed up to this point possess essentially no amyloid-like β-structure according to their AWN-ThT values (Fig. 2k, red square). Within this initial 10 h period EM images show a transition from initial spherical oligomers (2 h, Fig. 2h) to filaments (5 h, Fig. 2i). At around 10 h monomer concentrations once again begin to decrease, and this is accompanied by a gradual increase in the AWN-ThT fluorescence (Fig. 2k). At about 90 h there is a plateau in both the monomer loss curve and the AWN-ThT curve (Fig. 2k), suggesting that the assembly reaction has come to a final equilibrium position. It is important to note that the very gradual rise in AWN-ThT is not necessarily expected. If the onset of further aggregation at 10 h involved formation of a homogeneous suspension of mature amyloid as the only aggregate present, the AWN-ThT should have immediately climbed to a value at or near that of mature fibrils as seen at 120 h. That this did not happen suggests a very gradual transition within a heterogeneous suspension of aggregates through a series of species with ever increasing levels of amyloid-like β-structure (Discussion). CD spectroscopy of reaction mixtures cannot distinguish between monomers and aggregates in suspension, but does confirm a gradual increase in β-structure in the entire reaction mixture from 10 h to the end of the reaction (Fig. 2j; Supplementary Fig. 3). Importantly, total α-helix content of the aggregation reaction increases to a maximum value in the first 10 h of the reaction (shaded grey bar), then decays sharply over the next 10–20 h (Fig. 2j). This rise and fall of α-helix content, which perfectly coincides with the ThT-negative phase of aggregation (Fig. 2k), is similar to what was previously observed for spontaneous assembly of Aβ itself^16^.

### Behaviour of K_2_Q_23_K_2_Aβ_30–40_

**Figure 3:**
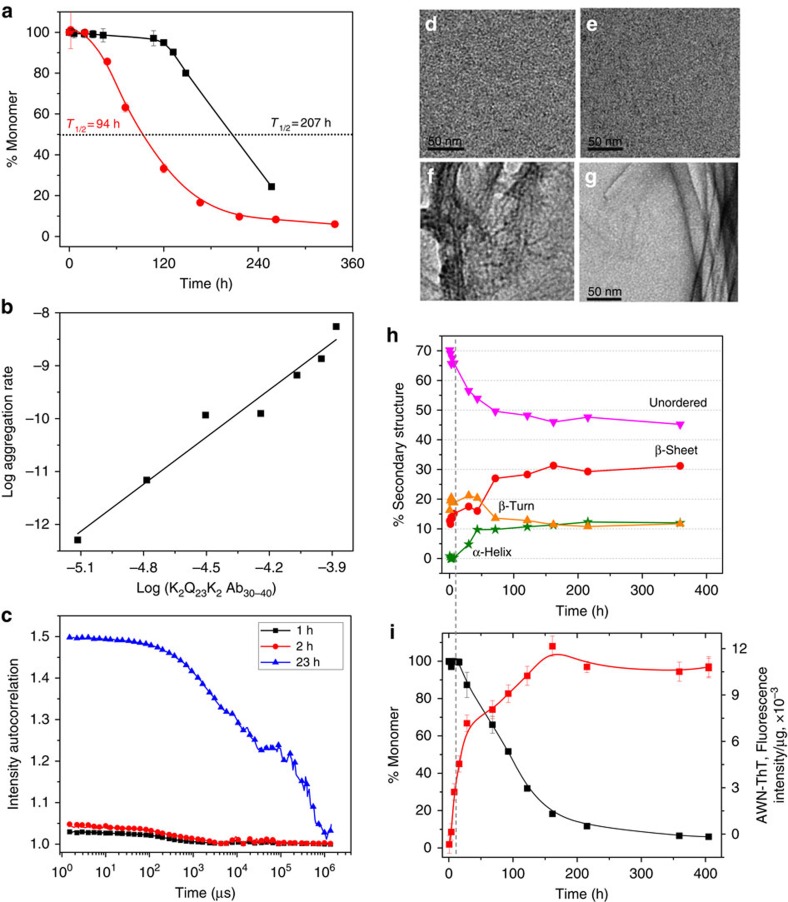
Aggregation of K_2_Q_23_K_2_Aβ_30–40_. (**a**) Aggregation assessed by disappearance of monomer for 37 °C incubation in PBS of 108 μM K_2_Q_23_K_2_Aβ_30–40_ (red circle) compared with 103 μM K_2_Q_23_K_2_ (black square). (**b**) Log–log plot of initial aggregation rates of K_2_Q_23_K_2_Aβ_30–40_ at various starting concentrations (Table 1 and Supplementary Fig. 1b). (**c**) Raw DLS data at different incubation times in the incubation of K_2_Q_23_K_2_Aβ_30–40_ at 112 μM in PBS at 37 °C (data indicated no oligomeric particles at 1–2 h and no interpretation at 24 h due to large highly scattering particles). (**d**–**g**) TEM images (scale bar, 50 nm) from the reaction described for **c** (**d**, 1 h; **e**, 2 h; **f**, 23 h; **g**, 432 h). (**h**) Time-dependent changes in secondary structure content from CD analysis (Supplementary Fig. 3b) of the reaction described in **c** (360 h, anti-parallel β-sheet=31%, parallel β-sheet=0%). (**i**) Aggregation kinetics of the reaction described in **c** as assessed by changes in monomer concentration (black square) and aggregate weight-normalized ThT fluorescence (AWN-ThT) (red square). The grey dashed line spanning **h** and **i** highlights the time just before substantial sedimentable aggregate begins to form; at this time, weight-normalized ThT for existing aggregates is already high, and total α-helix content is negligible.

It is a common observation that Aβ_40_ is much less amyloidogenic than Aβ_42_ (refs 15, 39). To test if our model system might replicate this trend, we studied the K_2_Q_23_K_2_Aβ_30–40_ peptide. For peptides incubated at ∼100 μM, we found a *t*_½_ for spontaneous aggregation of K_2_Q_23_K_2_Aβ_30–40_ of 94 h (Fig. 3a, red circle), in contrast to 207 h for K_2_Q_23_K_2_ (Fig. 3a, black square). This rate increase of only about twofold is markedly smaller than the 100-fold increase conferred by the Aβ_30–42_ segment (Fig. 2a). In addition, the slope of the log–log plot for K_2_Q_23_K_2_Aβ_30–40_ is 2.9 (Fig. 3b, Table 1), inconsistent with an oligomer-mediated amyloid assembly reaction^22^ but consistent with a classical nucleated growth polymerization reaction with n*=1 (ref. 8). (This nucleus size contrasts with that of K_2_Q_23_K_2_ itself, which gives n*=4 (Table 1)^8^. However, small structural changes in this transitional repeat length range^8^ have been shown to greatly affect the size of the critical nucleus^40^.) Like K_2_Q_23_K_2_ (ref. 8), and unlike HTT exon1-like fragments^22^ and K_2_Q_23_K_2_Aβ_30–42_ (Fig. 2), K_2_Q_23_K_2_Aβ_30–40_ incubated at 112 μM exhibits no evidence by EM (Fig. 3d,e) or DLS (Fig. 3c) of any particles prior to the onset of formation of sedimentable, fibril-like particles (Fig. 3f) at ∼1 day incubation time. These trends are mirrored in the AWN-ThT values that rapidly increase around 10 h, when sedimentable aggregates just begin to appear (Fig. 3i, dashed line). In contrast to K_2_Q_23_K_2_Aβ_30–42_, K_2_Q_23_K_2_Aβ_30–40_ exhibits no evidence of α-helix in this time frame, but rather undergoes small increases in β-sheet and β-turns (Fig. 3h). At 5 days, when the aggregation reaction is 70% complete by the sedimentation assay (Fig. 3i), the β-sheet plus β-turn content of the reaction mixture reaches about 40%. This implies that the mature amyloid fibrils that have formed by five days (as evidenced by the nearly maximized AWN-ThT value) should possess about 60% of the maximum possible β-structure, consistent with a peptide whose 23 Gln residues account for 61% of the total residues. Together these data paint a picture of a polyQ-containing peptide that aggregates very much like simple polyQ peptides. Thus, the two C-terminal residues of Aβ_30–42_ are responsible for a qualitative difference in aggregation rates and aggregation mechanisms in these polyQ fusion peptides.

### Behaviour of K_2_Q_23_Aβ_25–42_

**Figure 4:**
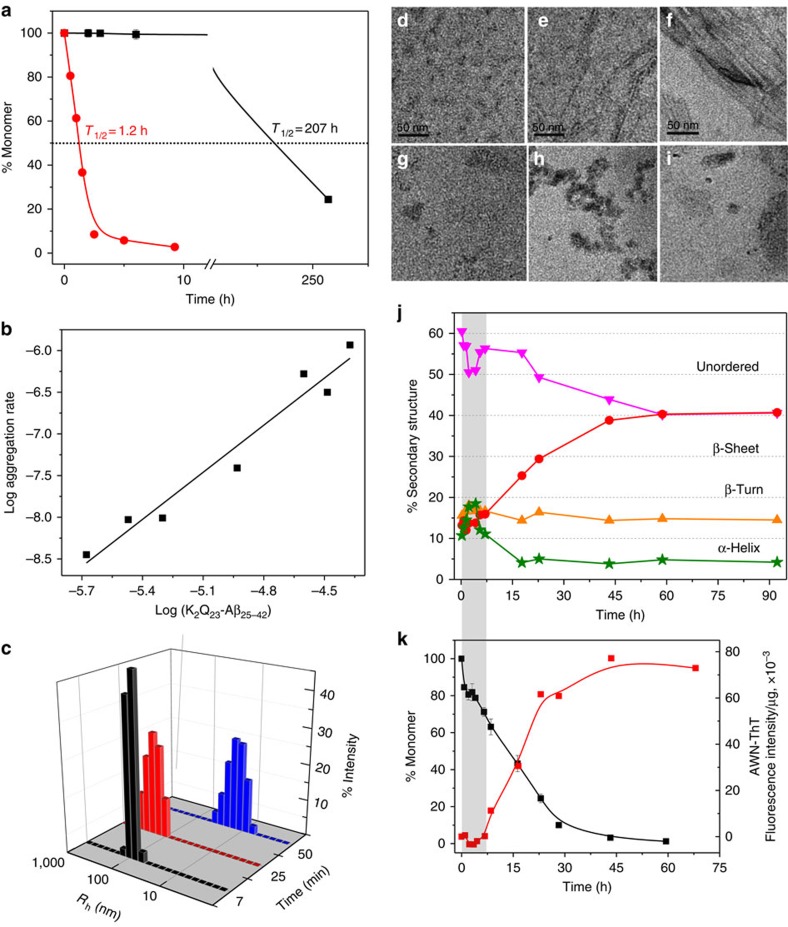
Aggregation of K_2_Q_23_Aβ_25–42_. (**a**) Aggregation assessed by disappearance of monomer for 37 °C incubation in PBS of 42 μM K_2_Q_23_Aβ_25–42_ (red circle) compared with 103 μM K_2_Q_23_K_2_ (black square). (**b**) Log–log plot of initial aggregation rates of K_2_Q_23_Aβ_25–42_ at various starting concentrations (Table 1 and Supplementary Fig. 1c). (**c**) Particle histograms from DLS analysis at different incubation times of 25 μM K_2_Q_23_Aβ_25–42_ in PBS at 37 °C (Supplementary Fig. 2b). (**d**–**i**) TEM images (scale bars, 50 nm) of incubation of K_2_Q_23_Aβ_25–42_ at 37 °C in PBS at either 25 μM (**d**,**e** 60 min; **f**, 1,200 min) or 3.4 μM (**g**, 15 min; **h**, 45 min; **i**, 2.7 h). (**j**) Time-dependent changes in secondary structure content from CD analysis (Supplementary Fig. 3c) of reaction mixtures of the incubation of 3.4 μM K_2_Q_23_Aβ_25–42_ at 37 °C in PBS (92 h, anti-parallel β-sheet=41%, parallel β-sheet=0%). (**k**) Aggregation kinetics of the reaction described in **j** as assessed by changes in monomer concentration (black square) and aggregate weight-normalized ThT fluorescence (AWN-ThT) (red square). The grey-shaded bar spanning **j** and **k** highlights the time domain in which the α-helix content increases while ThT fluorescence is nil.

We found that K_2_Q_23_Aβ_25–42_ behaves similarly to K_2_Q_23_K_2_Aβ_30–42_, while exhibiting somewhat faster amyloid growth rates. Thus, even while incubated at less than half the starting concentration, 42 μM K_2_Q_23_Aβ_25–42_ exhibits a *t*_½_ for aggregation that is ∼200 times lower than that for 103 μM K_2_Q_23_K_2_ (Fig. 4a). Like K_2_Q_23_K_2_Aβ_30–42_, it appears to form amyloid via a pathway featuring oligomeric intermediates, as indicated by its log–log slope of 1.9 (apparent n*=−0.1) (Fig. 4b, Table 1), and by the appearance of light scattering (Fig. 4c), oligomeric (Fig. 4d,e,g–i) species at early time points. Similarly to K_2_Q_23_K_2_Aβ_30–42_, even at a very low concentration of 3.4 μM it exhibits formation within the first 5 h of sedimentable (Fig. 4k, black square), oligomeric (Fig. 4g–i) aggregates that are ThT-negative (Fig. 4k, red square) and rich in α-helix (Fig. 4j). After a rapid drop in α-helix and gain in β-structure that is complete at ∼7 h (Fig. 4j,k; grey bar), a second phase of the aggregation reactions begins featuring steadily increasing AWN-ThT (Fig. 4k) and β-sheet (Fig. 4j). Secondary structure compositions, sedimentable monomer, and AWN-ThT all reach plateaus at ∼45–60 h (Fig.4j,k). The small 8% amplitude (compared with a 10% baseline) of biphasic α-helical changes reaching a maximum at 6–8 h is likely entirely associated with the oligomeric fraction. Since the Aβ segment accounts for 40% of the K_2_Q_23_Aβ_25–42_ residues, and since 20% of the molecules in the reaction mixture are assembled into sedimentable oligomers at 6–8 h (Fig. 4k, black square), the entire reaction mixture at this time should exhibit 8% α-helix attributable to transient oligomer formation—as observed.

### Robustness of α-helix detection

The behaviour of these three peptides is consistent with a model in which the Aβ_30–42_ segment, but not the Aβ_30–40_ segment, triggers formation of amyloid fibrils by mediating rapid and transient formation of α-helical oligomers. The data supporting transient α-helix, however, derives from deconvolution of CD spectra, which can be prone to a number of possible fitting artefacts, such as from inaccurate concentration determination and from shifts in CD spectra due to light scattering (LS). It is therefore important to examine the robustness of the evidence for transient α-helix in the hybrid peptides.

We believe our method for analytical HPLC determination of peptide concentrations^41^ used to set-up the aggregation reactions is highly accurate. Even if the concentrations of the starting reaction mixtures were imprecise, however, it could not produce the kind of waxing and waning of α-helix we see in the peptide aggregation time courses, since the same (starting) concentration value is used to calculate molar ellipticities at all reaction time points.

Although severe LS can distort CD curves and make deconvolution unreliable, we also do not believe LS is producing artifacts in our experiments. First, we have observed that on a weight concentration basis mature polyQ amyloid gives relatively low turbidity/LS compared with other amyloids. In fact, it is sufficiently low that it allows accurate deconvolution of secondary structure in mature polyQ amyloid that reveals its independently characterized^26^ anti-parallel β-sheet structure^42^. Similarly, the anti-parallel basis of the polyQ cores is consistently revealed in the mature amyloids of the three hybrid Aβ-polyQ peptides in spite of some LS in the sample (Figs 2, 3, 4, legends). Second, the burst of α-helix observed here occurs very early in the aggregation reactions at times when LS is much lower than in the final reaction mixtures. Third, increases in β-structure estimated from CD deconvolution closely parallel increases indicated by AWN-ThT values (Figs 2, 3, 4). Fourth, estimates of the percentages of β-structure in the mature amyloids are very consistent with calculated percentages based on independently determined weight concentrations of aggregates and percentages of peptide sequences found in the polyQ amyloid cores. Fifth, samples of the freshly dissolved Aβ C-terminal fragment K_4_G_2_Aβ_30–42_ at intermediate concentrations exhibit a mixture of disorder and α-helix in the CD, consistent with the ability of C-terminal Aβ peptides to readily access mixtures of disordered monomer and α-oligomers (Supplementary Fig. 4). Sixth, at higher concentrations where α-oligomer formation leads to nucleation of amyloid-like structure, K_4_G_2_Aβ_30–42_ exhibits similar mixtures of α-helix and β-sheet as assessed by both CD and FTIR (Supplementary Fig. 4), consistent with accurate assessment of secondary structure levels from CD deconvolution even in the presence of LS. Seventh, CD deconvolution of this peptide is so accurate that it reveals that the β-sheet component is anti-parallel β-sheet, as expected from FTIR analysis of mature Aβ_31–42_ fibrils (Supplementary Fig. 4). Eighth, FTIR spectra of inclusion bodies from bacterial expression of Aβ indicate substantial α-helical content, and solid state NMR spectrometry and hydrogen–deuterium exchange indicate coiled coil helix involving the Aβ C terminus^43^.

### Aggregate structure

**Figure 5:**
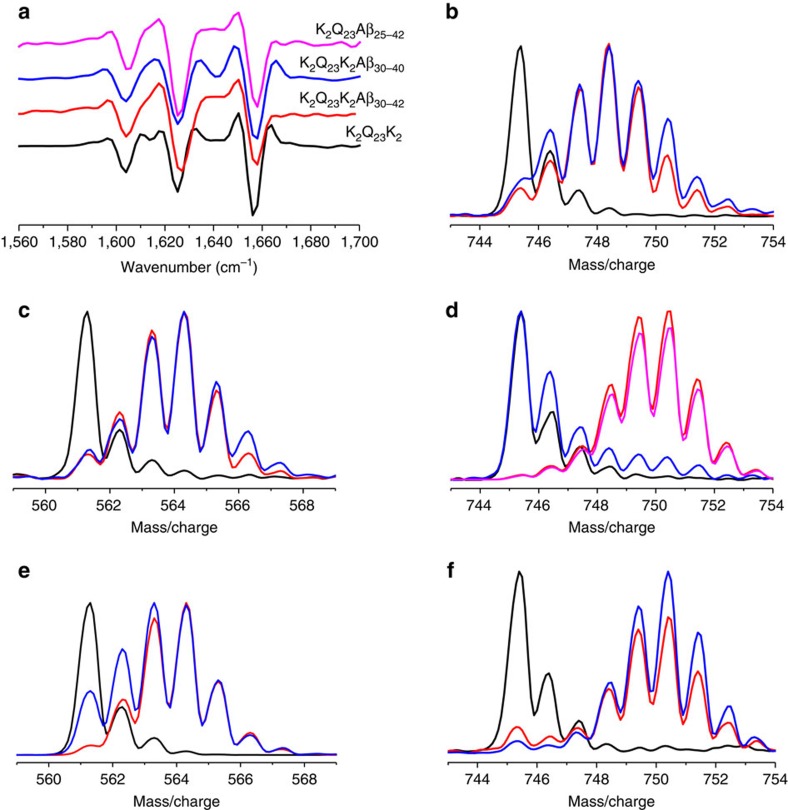
Structural features of mature amyloid products. (**a**) Second derivative FTIR spectra of amyloid products showing the triplet of bands characteristic of polyQ amyloid^23^. (**b**–**f**) Mass/charge distribution from HX-MS analysis of the C-terminal fragment generated by pepsin cleavage between Leu^34^ and Met^35^ of various peptides (protonated monomer, black line; deuterated monomer, red line; Gdn-HCl dissolved deuterated fibrils, blue line; aggregates formed in presence of K_10_G_2_Aβ_31–42_, magenta line): (**b**). K_2_Q_23_K_2_Aβ_30–42_; (**c**) K_2_Q_23_K_2_Aβ_30–40_; (**d**) Aβ_42_; (**e**) Aβ_40_; (**f**) K_2_Q_23_Aβ_25–42_.

According to our hypothesis and designs of the peptide models, we expected the Aβ segments of the hybrid peptides to not be involved in amyloid core formation. FTIR spectroscopy showed that the mature amyloid fibrils of all three hybrid peptides exhibit typical polyQ amyloid second derivative spectra (Fig. 5a), and CD deconvolution showed that the β-sheets in the mature fibrils are anti-parallel (Figs 2, 3, 4 legends), as expected for a polyQ core. Since the typical α-helix band in FTIR overlaps the characteristic polyQ amyloid band at ∼1,657 cm^−1^ (ref. 23), it is not expected that we might observe any α-helical structure in these fibrils that might be analogous to the HTT^NT^ α-helix in HTT exon1 fibrils^25^. To address whether a portion of the Aβ segment might be involved in stable β-structure in the fibrils, we used hydrogen–deuterium exchange evaluated by mass spectrometry (HX-MS). As shown previously^18^, in mature amyloid fibrils of Aβ_42_, the C-terminal fragment starting at residue 35 resists exchange, leading to a 35–42 fragment that, after exposure of fibrils to D_2_O, has a mass distribution (Fig. 5d, blue) that is much more like that from protonated Aβ_42_ monomers (Fig. 5d, black) and quite unlike the fragment from fully deuterated Aβ_42_ monomers (Fig. 5d, red). This behaviour contrasts with that of Aβ_40_ fibrils exposed to D_2_O, which release a C-terminal 35–40 fragment that is essentially fully exchanged (Fig. 5e) (ref. 18). We found that in the mature fibrils of all three polyQ-Aβ hybrid peptides, the 35–42/35–40 fragments released from fibrils after exchange (blue) resemble the corresponding fragment from deuterated monomers (red), and therefore are essentially fully exchanged (Fig. 5b,c,f). This suggests that the Aβ segments are not involved in amyloid core structure. The absence of a large amount of β-structure within the Aβ segment of the polyQ-Aβ hybrid fibrils is consistent with the total β-structure content of about 50% seen in the CD spectra of the final amyloid suspensions (Figs 2j, 3h and 4j) of peptides containing about 50% Gln residues. Given the ability of Aβ C-terminal peptide fragments ending at residue 42 to assemble into amyloid fibrils (Supplementary Fig. 4), it is remarkable that the same sequence appended to polyQ resists adopting highly protective β-secondary structure within the Aβ component in these hybrid peptide fibrils. This is may be due to topological or other structural restrictions imposed by details of the polyQ amyloid core.

We also addressed whether the Aβ appendages of these hybrid peptides provide any additional stability to the Q_23_ amyloid cores, in analogy to the stabilizing effect of HTT^NT^ on HTT exon1 amyloid^27^. Stabilities were assessed by determining the concentration of monomer present when the amyloid assembly reaction reaches equilibrium (Supplementary Fig. 5)^41^. The lower the *C*_r_ value, the more stable the fibrils with respect to monomers in solution. The results (Table 1) indicate some interesting trends. First, we found that the *C*_r_ value of 3.6 μM for K_2_Q_23_K_2_Aβ_30–40_ is essentially the same as the value of 3.0 μM previously reported for K_2_Q_23_K_2_, suggesting that the Aβ_30–40_ sequence does not provide stabilizing interactions within the fibrils. This is in stark contrast to the Aβ peptides ending at residue 42. The *C*_r_ for K_2_Q_23_K_2_Aβ_30–42_ is 0.42, or about sevenfold lower than that of K_2_Q_23_K_2_, and the *C*_r_ for K_2_Q_23_Aβ_25–42_ is 0.08, almost 40-fold lower than that of K_2_Q_23_K_2_. Interestingly, these stabilizations by Aβ fragment flanking sequences are similar to those obtained for Q_23_ peptides in HTT exon1 flanking sequence contexts (Table 1)^27^, and the ∼9-fold difference between *C*_r_ values of K_2_Q_23_K_2_Aβ_30–40_ and K_2_Q_23_K_2_Aβ_30–42_ is remarkably similar to the ∼10-fold difference in the C_r_ values of Aβ_40_ (1.06±0.18 μM;^38^) and Aβ_42_ (0.11±0.01 μM;^18^) fibrils. These similar relative stabilities of mature fibrils are achieved in spite of radically different structural roles for the C-terminal segments in fibrils of Aβ compared with polyQ-Aβ hybrids.

### A probe of mechanism using the Aβ_31–42_ fragment

In the HTT system, addition of isolated HTT^NT^ peptides to aggregation reactions modestly delays the onset of amyloid formation by HTT exon1-like fragments^28^. This appears to involve dilution of local polyQ concentrations when mixed α-oligomers are formed between HTT exon1-like fragments and the HTT^NT^ sequence (compare Fig. 1d with Fig. 1c). We set out to investigate whether an appropriate Aβ C-terminal fragment might act similarly to delay aggregation onset by the hybrid Aβ-polyQ peptides. First, we attempted to improve the poor kinetic solubility of the Aβ_31–42_ fragment^44^ by adding a series of Lys residues to its N terminus to generate K_10_G_2_Aβ_31–42_ (Fig. 1f).

On physical characterization of this peptide, we found that at all concentrations tested, K_10_G_2_Aβ_31–42_ incubated alone leads to rapid formation of a small percentage of sedimentable aggregates, after which there is a concentration-dependent further aggregation that is negligible, over the first 75 h, at concentrations of 10 μM or less (Supplementary Fig. 6a). At 22 μM after 24 h, aggregate suspensions exhibit negligible ThT fluorescence (Supplementary Fig. 6b), overall 22% α-helix (Supplementary Fig. 6c) and a uniform distribution of spherical oligomers of 20–30 nm radius (Supplementary Fig. 6d; 2 h). After 24 h, the AWN-ThT signal increases steadily until reaching a maximum at 7 days (Supplementary Fig. 6b), suggesting a homogeneous β-aggregate which, at 23 days, is completely fibrillar (Supplementary Fig. 6d; 23 days). Since after 35 days ∼13 μM of monomer remain in the reaction mixture (Supplementary Fig. 6b), this peptide's fibrils likely have a *C*_r_ value in the 10–13 μM range. The data suggest that K_10_G_2_Aβ_31–42_ itself undergoes an oligomer-mediated amyloid initiation mechanism that is unfavourable at concentrations of 10 μM or less but which advances slowly at ∼20 μM. This solubility behaviour revealed a limited window of concentrations that can be used to test K_10_G_2_Aβ_31–42_ as an inhibitor, above which the inhibitor itself forms fibrils.

**Figure 6:**
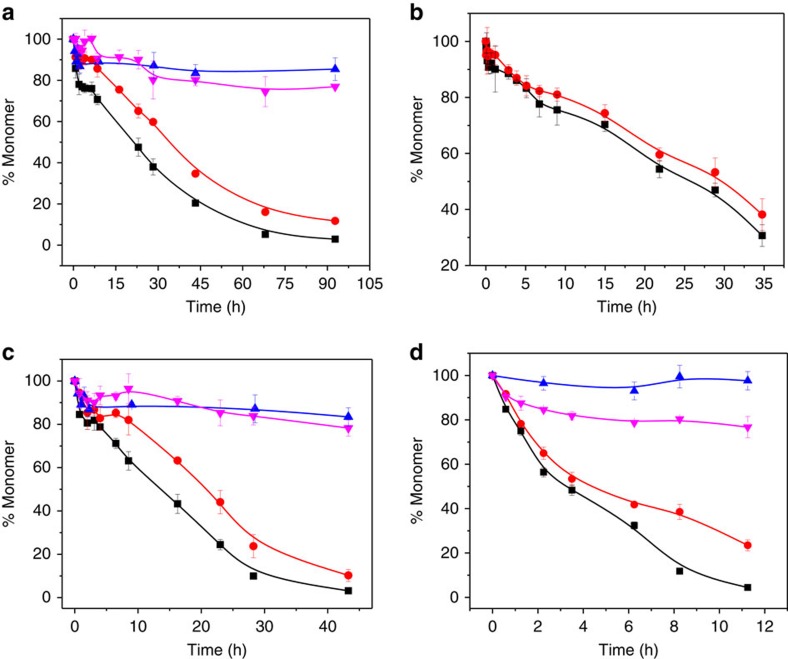
Inhibition of spontaneous amyloid formation by K_10_G_2_Aβ_31–42_. Results of sedimentation-HPLC assays on mixtures of Aβ-related peptides and K_10_G_2_Aβ_31–42_. (**a**) K_2_Q_23_K_2_Aβ_30–42_ (6.4 μM) and K_10_G_2_Aβ_31–42_ (7.6 μM). (**b**) K_2_Q_23_K_2_Aβ_30–40_ (16.7 μM) and K_10_G_2_Aβ_31–42_ (23 μM). (**c**) K_2_Q_23_Aβ_25–42_ (3.9 μM) and K_10_G_2_Aβ_31–42_ (6.9 μM). (**d**) Aβ_42_ (3.1 μM) and K_10_G_2_Aβ_31–42_ (8.3 μM). Symbols: amyloidogenic peptide alone (black square); K_10_G_2_Aβ_31–42_ alone (pink inverted triangle); mixture of amyloidogenic peptide (red circle) and K_10_G_2_Aβ_31–42_ (blue triangle).

**Figure 7:**
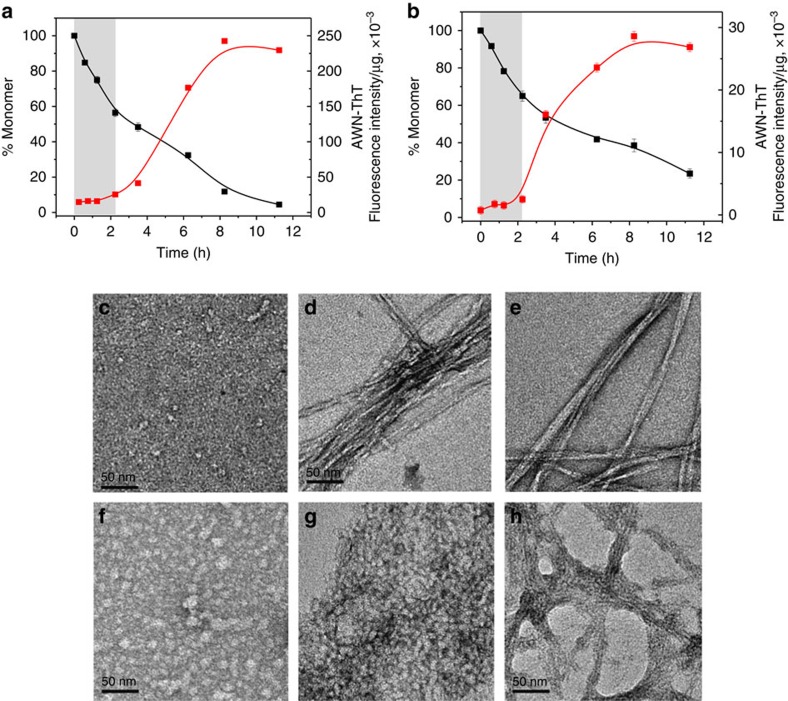
Effect on Aβ_42_ amyloid formation by K_10_G_2_Aβ_31–42_. (**a**,**b**) Kinetics of Aβ_42_ self-assembly at 37° in PBS, as assessed by decreases in monomer concentration (black square) and changes in aggregate weight-normalized ThT fluorescence (AWN-ThT) (red square), in reactions of 2.7 μM Aβ_42_ alone (**a**) or 3.1 μM Aβ_42_ mixed with 8.3 μM K_10_G_2_Aβ_31–42_ (**b**). (**c**–**h**). TEM images (scale bars, 50 nm) of reaction time points of 7.4 μM Aβ_42_ incubated alone (**c**–**e**) or with 11.5 μM K_10_G_2_Aβ_31–42_ (**f**–**h**), with time points at 1 h (**c**,**f**), 4 h (**d**,**g**) and 24 h (**e**,**h**).

We found that K_10_G_2_Aβ_31–42_ induces a modest delay in the onset of aggregation in both K_2_Q_23_K_2_Aβ_30–42_ and K_2_Q_23_Aβ_25–42_, but not K_2_Q_23_K_2_Aβ_30–40_. Thus, at molar ratios of 1.2 and 1.8, K_10_G_2_Aβ_31–42_ shifts the aggregation curves of K_2_Q_23_K_2_Aβ_30–42_ and K_2_Q_23_Aβ_25–42_ by 15 h (Fig. 6a) and 7 h (Fig. 6c), respectively. Consistent with our model, these delays are associated with initial formation of sedimentable aggregates that exhibit very low AWN-ThT signals (Supplementary Fig. 7a,d). The ability to delay, but not eliminate, onset of amyloid formation is very similar to the action of HTT^NT^ against HTT exon1-like peptides^28^. In contrast to the hybrid peptides ending at Aβ residue 42, a 1.4:1 ratio of K_10_G_2_Aβ_31–42_ to K_2_Q_23_K_2_Aβ_30–40_ neither significantly shifts the latter's aggregation curve (Fig. 6b) nor eliminates the immediate ThT-positive content of its aggregates (Supplementary Fig. 7b). Although the inhibition we observed in these experiments was modest, the observed inhibition further supports a role for α-helical, ThT-negative oligomers in fibril formation by K_2_Q_23_K_2_Aβ_30–42_ and K_2_Q_23_Aβ_25–42_.

### Spontaneous aggregation of Aβ_42_

Previously we were unable to tease out details of Aβ_42_ amyloid assembly at a starting concentration of 10 μM due to rapid onset of amyloid formation^18^. Here we used a substantially lower concentration that is at the same time still above the peptide's *C*_r_ of 0.11 μM (ref. 18) leading to a reaction that exhibits a good lag phase in the development of AWN-ThT, confirming an early non-β oligomeric intermediate (Fig. 7a). Under these conditions, we inquired whether K_10_G_2_Aβ_31–42_ might also inhibit spontaneous amyloid formation by Aβ_42_. In a reaction of 3.1 μM Aβ_42_ and 8.3 μM K_10_G_2_Aβ_31–42_, however, we observed little effect on aggregation kinetics as assessed by either the sedimentation-HPLC assay or by AWN-ThT (Fig. 7b). At the same time, Aβ_42_ grown in the presence of K_10_G_2_Aβ_31–42_ showed a markedly (∼10-fold) lower final AWN-ThT value (Fig. 7b), suggesting a substantially different end-stage aggregate morphology compared with normally produced fibrils. We confirmed this in a repeat study at 7.4 μM Aβ_42_ plus 11.5 μM K_10_G_2_Aβ_31–42_, which showed—compared with Aβ_42_ alone—larger initial oligomers (Fig. 7f versus Fig. 7c), a prolonged protofibril stage (Fig. 7g versus Fig. 7d), and a different mature fibrillar morphology (Fig. 7h versus Fig. 7e). The different structure in the mature fibrils was confirmed by FTIR, which showed a new, anti-parallel β-sheet band (Supplementary Fig. 8), and by HX-MS. For global HX, Aβ_42_ aggregated alone showed a typical^18^ incorporation of 14.8±1.2 deuteriums, while Aβ_42_ aggregates grown in the presence of K_10_G_2_Aβ_31–42_ showed 25.6±1.2 deuteriums. Many of the ∼11 newly unprotected amide hydrogens in the latter are located in the peptide's C terminus, as shown by segmental HX-MS (Fig. 5d, magenta line). We confirmed that the aggregates subjected to FTIR and HX-MS contained only Aβ_42_ (and not K_10_G_2_Aβ_31–42_ inhibitor) by HPLC of dissolved aggregates (Supplementary Fig. 8c).

## Discussion

**Figure 8:**
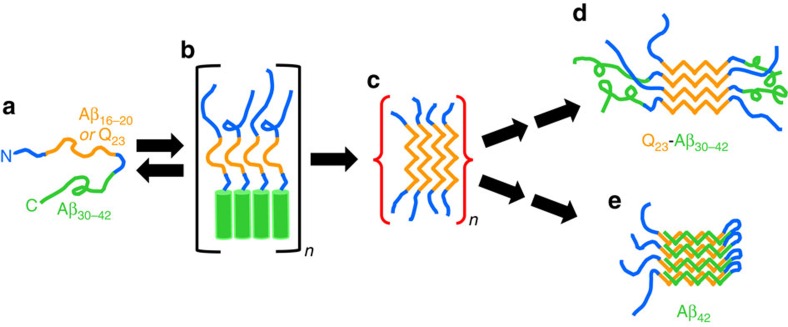
Model for a modular role for the Aβ C terminus in amyloid initiation. Monomeric peptides containing an Aβ C terminus ending at residue 42 (**a**) undergo rapid formation of a small, metastable population of oligomers held together by packing of α-helical Aβ C termini (**b**). The resulting high local concentration of amyloidogenic segments in these oligomers overcomes the concentration barrier leading to formation of β-rich amyloid intermediates (**c**). These progress, through a poorly defined combination of structural rearrangements and seeded elongation, to mature amyloid (**d**,**e**). For polyQ-Aβ hybrids, the resulting amyloid has an anti-parallel β-sheet polyQ core decorated with non-β Aβ segments that appear to interact within the fibril (**d**). For Aβ_42_, the resulting amyloid adopts parallel, in-register β-sheet that includes the Aβ C-terminal segment (**e**). Aβ C terminus (green), amyloidogenic polyQ and Aβ_16–20_ (orange), hydrophilic segments (see Fig. 1f) (blue).

Our results are consistent with a HTT exon1-like amyloid assembly mechanism for the Q_23_-Aβ_42_ hybrid peptides (Fig. 8) that features rapid formation of transient oligomeric intermediates held together by non-amyloid-type contacts involving the Aβ segments. The resulting high local concentration of the tethered Q_23_ segments overcomes the high-concentration barrier for polyQ amyloid nucleation to initiate fibril growth. Although it can be challenging to obtain accurate secondary structures by deconvolution of CD spectra of protein aggregates, there are a number of reasons, outlined in the Results section, to trust the experimental indications that these transient oligomers are held together as bundles of α-helices composed of the Aβ C terminus.

The primary nucleation phase for amyloid formation by Aβ peptides is particularly important to understand for two reasons. First, and foremost, without primary nucleation amyloid assembly can never occur. Second, non-fibrillar, oligomeric intermediates forming early in Aβ amyloid assembly have been implicated as particularly toxic species associated with the mechanism of Alzheimer's disease^45^. Through the design and study of Aβ-polyQ hybrid peptides we specifically investigated the early steps of Aβ assembly leading to the initiation of amyloid formation. By replacing the LVFF segment of Aβ with a polyQ segment we expected the amyloid initiation phase of Aβ itself to be replicated, but the downstream consolidation of additional amyloid structure in the C terminus of Aβ to not to be replicated. In fact, the data on the three hybrid peptides support a model for the earliest steps of Aβ_42_ assembly that is largely identical to what we see for the two hybrid peptides ending at position 42 of Aβ. Thus, for both 6 μM K_2_Q_23_K_2_Aβ_30–42_ and for 3.4 μM K_2_Q_23_Aβ_25–42_ we observe formation of sedimentable aggregates that exhibit no ThT signal (Figs 2k and 4k) in exact analogy to the ThT-negative aggregates formed in the early phase of Aβ_42_ assembly (Fig. 7a). For the reactions of the two hybrid peptides this ThT-negative phase occurs precisely when CD analysis of the reaction mixtures reveal transient formation of α-helix (Figs 2j and 4j) and transient formation of spherical oligomers (Figs 2h and 4h). Similarly, transient α-helix^16^ and formation of spherical oligomers^2^ (Fig. 7c) have been observed early in the self-assembly of Aβ peptides. As expected for the proposed nucleation mechanism^28^, we also observed modulation of the self-assembly of both Aβ_42_ (Fig. 7) and the hybrid peptides (Fig. 6) by the presence of an Aβ_31–42_-related peptide. Finally, the markedly different assembly kinetics of the model hybrid peptides K_2_Q_23_K_2_Aβ_30–40_ (Fig. 3a) and K_2_Q_23_K_2_Aβ_30–42_ (Fig. 2a) beautifully reproduce the well-known kinetic difference in the amyloid assembly of Aβ_40_ and Aβ_42_ (ref. 39), while suggesting that these two highly related Aβ peptides undergo substantially different mechanisms for initiation of amyloid formation.

Thus, our results for Aβ-polyQ hybrids support an analogous nucleation mechanism for longer versions of Aβ itself, in which the Aβ C terminus initially plays a similar role of mediating α-helical oligomer formation before this segment later becomes swallowed up in the amyloid core. Based on solution NMR analysis of Aβ_42_ monomers that found no evidence of any residual secondary structure in this peptide^14^, Bax and colleagues recently proposed that early aggregation intermediates must be assembled via hydrophobically driven self-interactions of peptide segments in irregular structure. While our data on Aβ_42_ concur that the first formed oligomers do not involve amyloid-like β-structure^18^ (Fig. 7), based on the polyQ-Aβ hybrid data provided here as well as previous data on Aβ assembly^16^ we think it is likely that the early oligomers are clusters of α-helical elements. In fact, there is a precedent in our HTT exon1 results for a peptide exhibiting very little α-structure as a monomer that nonetheless forms robust α-helix rich assembly intermediates^22^,^23^,^28^. Such counter-intuitive phenomena seem possible, for example, if there are levels of helical peptides in the monomer ensemble that are below NMR detection limits but sufficient to support helical bundle assembly. Alternatively, helical bundles might develop by concerted mechanisms in which peptides acquire helical structure during self-assembly^46^. In analogy to the proposed involvement of α-helix rich oligomers in an amyloid nucleation mechanism, some β-sheet rich globular proteins are known to acquire their native structures via α-helix-rich folding intermediates^47^,^48^.

Our data strongly suggest that the two C-terminal residues of Aβ_42_ enhance amyloid formation kinetics compared with Aβ_40_ by favouring the formation of α-helical oligomeric intermediates that serve as the phase within which a particularly efficient amyloid nucleation occurs. A separate segment of Aβ—probably centred in the LVFF region—acts as the driving amyloidogenic segment involved in this second step. In our polyQ-Aβ model peptides, the mature amyloid core structure appears to be confined to the polyQ segment, while the Aβ portion does not possess highly protective backbone H-bonded structure. We propose that in Aβ_42_ itself, a similar early β-intermediate is formed featuring amyloid-like structure only in the central region of the peptide including the LVFF segment (Fig. 8). Interestingly, a highly similar structural model featuring β-structure in the LVFF segment and α-structure in the C terminus (Fig. 1e) has been deduced for Aβ in inclusions bodies formed in bacteria, which lead to the suggestion that such a structure might be relevant to the normal amyloid assembly mechanism^43^. The mechanism and timing of the ‘finishing steps' required to move from such early β-intermediates to mature amyloid, however, are not immediately obvious.

Some insights into these finishing steps are offered by the AWN-ThT kinetics. In the model peptide K_2_Q_23_Aβ_25–42_, the period featuring the initial rise and fall of α-structure and the lag in ThT is over at 7 h, after which both total β-structure and AWN-ThT begin to rise inexorably (Fig. 4j,k). However, the subsequent trend in AWN-ThT values does not resemble that expected for a simple nucleated growth process. That is, in a classical nucleated growth polymerization mechanism, where the product of nucleus formation and initial elongation is immediately amyloid-like, the AWN-ThT is expected to jump to a high value immediately after nucleation (Supplementary Fig. 7b,e; Fig. 3i). In contrast, the AWN-ThT for K_2_Q_23_Aβ_25–42_ trends upward very slowly and gradually during amyloid assembly, beginning after α-structure is lost. Thus, although there is a rapid loss of α-helix from 4 to 8 h (Fig. 4j), the commensurate immediate gains in total β-structure (Fig. 4j) and AWN-ThT (Fig. 4k) are small and inconsistent with the formation of mature fibrils in the ∼40% of Aβ molecules that are aggregated at this time (Fig. 4k). In fact, AWN-ThT develops very slowly over the entire course of the reaction, only reaching a maximum value after ∼90% of the monomers have been consumed. At the same time, the coincidence between the sharp drop in α-helix and the onset of AWN-ThT at ∼7 h, in addition to the response by the hybrid peptides to the inhibitor K_10_G_2_Aβ_31–42_, supports the view that the α-intermediates play an early on-pathway role in amyloid assembly.

Amyloid assembly of Aβ_42_ itself follows a similar trend. In the first 2 h of incubation, before AWN-ThT begins to rise, ∼45% of monomers have been converted to aggregates (Fig. 7a). At the end of the AWN-ThT lag, there are no rapid bursts either in the rate of overall aggregation or in the magnitude of AWN-ThT as would be expected from a group-wise conversion to mature amyloid nuclei. Instead, from 2 to 8 h another 45% of the monomer pool is converted relatively slowly to the aggregate fraction, with a corresponding very gradual rise in AWN-ThT. There are several ways these data can be rationalized. It is possible that a small percentage of the conversion of α-oligomers marking the end of the null AWN-ThT phase might be associated with the formation of mature amyloid nuclei, whose elongation then takes place ‘under the radar' of the ensemble AWN-ThT fluorescence. This low level elongation activity might occur via growth supported either by the still substantial monomer pool (that is, a classical nucleated growth polymerization) or by some population of the multitude^18^ of oligomer types that persist after the decay of α-oligomers (that is, nucleated conformational conversion). Alternatively, the group-wise conversion of α-helical oligomers to aggregates with modest levels of β-structure might be the first in a series of group-wise changes, each of which is associated with a stabilizing increase in amyloid-like β-structure and therefore AWN-ThT, culminating in a final remodelling step into mature amyloid. The latter mechanism is consistent with reports that protofibrillar intermediates exhibit apparent β-structure in the same Aβ segments that are in β-sheet in mature fibrils^21^,^49^. Finally, the complex waxing and waning of various oligomeric intermediates might even be an off-pathway smokescreen masking the operation of a classical nucleated growth polymerization mechanism^10^—a mechanism that might become especially important only at lower Aβ concentrations.

In additional support of the model for how the Aβ C terminus mediates α-oligomer formation to initiate the amyloid assembly pathway, we found that K_10_G_2_Aβ_31–42_ delays onset of β-aggregation by K_2_Q_23_K_2_Aβ_30–42_ and K_2_Q_23_Aβ_25–42_ (which exhibit transient α-helix) but not by K_2_Q_23_K_2_Aβ_30–40_ (which does not). Against Aβ_42_ itself, K_10_G_2_Aβ_31–42_ has little effect on the kinetics of aggregation (Figs 6d and 7b). The reaction mixture passes through a stage with intermediate AWN-ThT values apparently derived from protofibril-like structures (Fig. 7g). The reaction proceeds to a final aggregate structure resembling the previously described D polymorph of Aβ_40_ fibrils^38^ in its EM morphology (Fig. 7h), low AWN-ThT value (Fig. 7b), and decreased protection against HX (Fig. 5d). Given our model for how K_10_G_2_Aβ_31–42_ might interact with Aβ_42_ during assembly, this result suggests that the ability of a peptide to assemble into a particular polymorphic form might be determined very early in the assembly process^50^, and thus that mechanisms such as the one investigated here might account for some polymorphic Aβ fibrils and not for others. The Aβ_42_ aggregates produced in the presence K_10_G_2_Aβ_31–42_ also resemble the assembly product of Aβ_42_ co-incubated with randomly generated peptides designed to form amphipathic α-helices^51^. This suggests a degree of promiscuity in the assembly of α-helical oligomeric intermediates that is very similar to what we previously described for HTT^NT^ inhibition of HTT exon1 aggregation^28^. C-terminal fragments of Aβ have been shown previously to inhibit Aβ cytotoxicity^52^, and these effects may well be related to the influence of such fragments on amyloid assembly kinetics and/or product structures.

Segmental mobility within intrinsically disordered proteins offers the theoretical possibility that through-space, long-range interactions between segments might play a role in defining intrinsically disordered protein global physical properties. Although it is difficult to establish such relationships experimentally, computations suggest that just such a process—rather than the modular effect invoked here—contributes to some aspects of the ability of HTT^NT^ to influence polyQ aggregation behaviour^53^. Further, the failure of one experimental test^54^ of the modular ‘local concentration' mechanism might be viewed as indirect support for such an integrative interaction mechanism. At the same time, the mechanistic alternative—the ability of a protein segment to behave as a quasi-independent unit to act in a modular manner to impact an aggregation mechanism—continues to attract strong experimental support. Besides the data on the Aβ C terminus reported here, supportive data have been previously reported for modular roles of HTT^NT^ (refs 22, 23, 24, 27, 28, 55, 56, 57), the Josephin domain of ataxin 3 (ref. 58) and the CRABP domain^59^ in protein aggregation mechanisms. The ability of HTT^NT^, as well as Aβ_30–42_ in a polyQ-Aβ hybrid, to bestow a large rate enhancement on spontaneous amyloid formation without itself being incorporated into the final amyloid core^26^, is relatively unusual in the amyloid literature. However, local concentration effects within rapidly formed non-β oligomers quite likely also contribute to some amyloid assembly reactions in which the segment mediating non-β oligomer assembly ultimately becomes incorporated into the amyloid core^60^, as suggested here and elsewhere^19^ for Aβ_42_.

In addition to implicating the C terminus of Aβ in the formation of on-pathway α-helical oligomeric intermediates that initiate amyloid formation, our data provide additional insights into the self-assembly process. For example, the relatively rapid initial formation of α-oligomers in both the polyQ-Aβ_42_ hybrids and in Aβ_42_ itself suggests that these initial aggregated structures are formed by a non-nucleated, energetically downhill process^6^. Our AWN-ThT data also suggest that α-intermediates do not directly convert *en masse* into rapidly elongating amyloid nuclei, but rather convert into immature protofibrillar intermediates exhibiting partial amyloid-like β-structure which continue to mature over much of the reaction coordinate in parallel with the continuing loss of monomeric Aβ from solution. That is, elongation seeded by mature fibrils, which normally is viewed as contributing the bulk of the magnitude of the ThT fluorescence of spontaneous amyloid growth, likely only accounts for the last ∼10% of the aggregation time course (Figs 2k, 4k and 7k). This would appear to rule out a major role for secondary nucleation processes^5^,^61^ in spontaneous Aβ amyloid assembly, at least under our experimental conditions. As recently suggested^18^, the AWN-ThT time course of Aβ_42_ reveals a rich menagerie of transient and rapidly interconverting aggregated species, from ThT-negative oligomers through a number of ThT-positive intermediates, any one of which might prove to be the critical aggregate responsible for AD toxicity. While β-oligomer forms of Aβ previously have received much attention in this regard, the possible contribution to cytotoxicity by more ephemeral α-oligomers remains to be explored.

## Methods

### Peptides

The peptides K_2_Q_23_K_2_Aβ_30–42_, K_2_Q_23_K_2_Aβ_30–40_ and K_2_Q_23_Aβ_25–42_ (calculated and observed monoisotopic masses of 4,670.467/4,670.466, 4,486.346/4,486.338 and 4,857.490/4,857.520, respectively) were obtained from Keck Biotechnology Center at Yale University ( http://info.med.yale.edu/wmkeck/). K_4_G_2_Aβ_31–42_ and K_10_G_2_Aβ_31–42_ (calculated and observed MWs of 1,768.27/1,768.35 and 2,537.31 and 2,537.40, respectively) were obtained from GenScript, Inc. These were obtained in crude form and purified as follows. After dissolution in 98% formic acid (Sigma) with sonication for 2 min in a bath sonicator, peptides were injected in a reverse phase HPLC column (9.4 × 250 mm Agilent Zorbax SB-C3) and eluted with 30–70% gradient of acetonitrile in water, with 0.05% trifluoroacetic acid (TFA, Pierce). Fractions with purities (LC-MS detector) exceeding 90% were pooled and lyophilized. Aβ_42_ (calculated and observed monoisotopic mass of 4,514.08/4,513.81) was obtained pre-purified from the Keck Center. Peptides were routinely disaggregated before every experiment using either the Gdn-HCl/SEC method^18^ (Aβ_42_) or the mixed TFA:hexofluoroisopropanol (HFIP, Acros Organics) method^41^ (all other peptides).

### Aggregation kinetics

Disaggregated peptide stock solutions were first checked for approximate concentration. All concentrations, for setting up the reaction and for assaying kinetics time points, were determined from integrated A_214_ HPLC (Agilent XDB-C18 4.6 × 50 mm column) peaks using standard curves individually constructed for each peptide from standard stock solutions calibrated via the A_215_ absorption^23^,^41^. Kinetics reaction mixtures were prepared by adding HPLC grade water to an aliquot of the peptide stock solution, plus a 1/9 volume of 10 × PBS buffer to give a final 1 × PBS. Sodium azide was added to 0.05% (w/v) and pH adjusted to 7.4. After filtration through a 0.2 μm filter unit (Anotop 10) to get rid of aggregates, the solution was held at 4 °C while the *t*=0 concentration was determined (∼30 min). The reaction mixture was moved to 37 °C and kinetics monitored by the sedimentation and ThT assays on reaction aliquots and by CD.

### Sedimentation assay

Duplicate time points were removed periodically, centrifuged 30 min at 436,000*g* followed by HPLC analysis of monomer concentration from the carefully removed supernatant^41^. Nucleation kinetics determination was carried out by evaluating initial time points of aggregation reactions with varied starting concentrations, as described^7^,^8^. Critical concentrations were determined from the average of the end point monomer concentrations of amyloid formation and dissociation reactions as described^41^. (See Supplementary Fig. 5).

### AWN-ThT assay

The ThT assay was performed by taking aliquots from the aggregation reaction mixtures containing 1.0 μg of total peptide and transferring to a 4 mm × 4 mm cuvette containing 20 μM ThT (Sigma) in 1 × PBS. Fluorescence was measured over a 2 min integration period using excitation wavelength at 445 nm (5 nm slit width) and emission at 489 nm (10 nm slit width) in a Fluoromax-4 spectrofluorometer (Horiba Jobin Yvon). AWN-ThT values were calculated for each time point by (a) subtracting the buffer+ThT blank, then (b) dividing this value by the mole fraction of peptides that were independently (by the HPLC sedimentation assay above) determined to be aggregated, according to the equation:


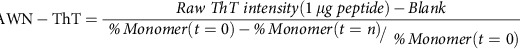


### Circular dichroism spectroscopy

Reactions to be studied in parallel by CD, sedimentation, and ThT were prepared in sufficient starting volume to allow aliquots to be taken for CD measurements. We chose to incubate these under exactly the same conditions as the other kinetics (rather than in the CD cuvette) in order to avoid differences due to possible surface effects. Aliquots were removed at different times and transferred to a 0.1 cm path length cuvette, and then the far-ultraviolet CD spectrum obtained on either a JASCO J-810 or OLIS 17 spectropolarimeter. Spectra were collected at 0.5 nm resolution with a scan rate of 100 nm min^−1^ (averaged over three scans) and corrected for the buffer signal. Secondary structure percentages were calculated from these spectra over the range 190–250 nm using the freely accessible algorithm BeStSel (bestsel.elte.hu), which includes independent basis spectra for both parallel and anti-parallel β-sheet^42^.

### Dynamic light scattering

DLS measurements were performed using a DynaPro plate reader (Wyatt Technology Corporation). An aliquot (∼80 μl) was transferred, after gentle mixing, from the ongoing aggregation reaction periodically to a fresh well of a 384-well microtitre plate, after which scattering data were obtained immediately. The intensity autocorrelation functions were analysed using a Dynamics V6 software to obtain the hydrodynamic radii (*R*_h_).

### Electron microscopy

Aliquots (∼8 μl) from the aggregation reaction at various time points were placed in a freshly glow-discharged carbon-coated 400 mesh size copper grids and allowed to adsorb for 90 s. The grid was washed with deionized water, stained with freshly filtered 8 μl of 1% (w/v) uranyl acetate for 30 s and then washed again with water. Each time, excess of sample, water and stain were blotted away with filter paper. Imaging was done using a Technai T12 microscope (FEI Company, Hillsboro, OR) operating at 120 kV with a magnification of × 30,000 and equipped with an Ultrascan 1000 CCD camera with post-column magnification of × 1.4.

### FTIR spectroscopy

Aggregates were collected at the end of aggregation reaction by centrifugation at 14,000 r.p.m. for 30 min. The pellet is washed once (PBS plus centrifugation), resuspended in ∼50 μl of 1 × PBS, and the suspension placed between two polished CaF_2_ windows using a BioCell module (BioTools, Inc.) on an ABB Prota-2 × MB 3000 FTIR instrument. Spectra were collected at 4 cm^−1^ resolution (average of 400 scans) and corrected for buffer absorption until a flat baseline was obtained in the 1,700–1,800 cm^−1^ region. Second derivative spectra for the amide-I region were calculated using PROTA software.

### Hydrogen exchange-mass spectrometry

All HX-MS data were analysed using an Agilent 1100 series single quadruple ESI mass spectrometer. Aggregates were washed and incubated in D_2_O and the exchange reactions analysed after an appropriate exchange time. Global HX was analysed by simultaneous dissolution and MS analysis of the exchange reaction using a T-tube front end to the MS^62^; data were not corrected for back-exchange. Segmental exchange was performed as described^63^. Briefly, fibrils were recovered from the exchange reaction by centrifugation (20,800*g*, 30 min), and in a 4 °C room to 20 μl ice-cold 8 M Gdn.HCl plus 0.1% formic acid was added to the pellets and mixed for 10 s, then 100 μl pepsin agarose (Sigma-Aldrich) in 0.1% cold formic acid was added and mixed for 10 s. The sample was immediately centrifuged to remove the immobilized pepsin (20,800*g*, 30 s) and the supernatant subjected to 4 °C LC-MS analysis.

### Reproducibility and data analysis

In amyloid kinetics analyses, the greatest determinant of lab-to-lab reproducibility lies in the details of peptide purity and source, preparation^8^, concentration determination^23^ and kinetics analysis^41^. In particular, it is critical to carry out a completely fresh disaggregation reaction on the peptide immediately before initiating each reaction, and to follow recommended disaggregation protocols^8^,^18^,^41^ exactly and completely. If a protocol must be modified or streamlined, it is critical, before carrying out detailed analyses, to determine that the modification does not compromise the desired monomeric state by introducing oligomeric forms or otherwise affect overall peptide behaviour.

For sedimentation and ThT kinetics, as well as kinetics underlying the n* and *C*_r_ determinations and inhibition kinetics studies, each plotted data point is derived from analysis of 2–3 independent reaction aliquots. Each aggregation study was independently carried out twice, with similar results obtained. For the time^2^ plots underlying the n* log–log plots, only the early phase of the aggregation reaction is considered, as described previously^8^,^41^; this corresponds to the first 2 h for Figs 2b and 4b, and the first 48 h for Fig. 3b. DLS autocorrelations were carried out on averages of at least 15 data acquisitions. Analysis of CD curves to produce time courses of secondary structural changes was accomplished on averages of at least three scans. EM images shown are representative of multiple similar images obtained under each experimental condition. FTIR and HX-MS analysis were conducted once on each end-stage aggregate.

Data sets for all experiments were analysed using Origin 7.5 software (OriginLab Corporation). The curves in sedimentation and ThT assay were fit to B-spline fitting function to guide the eye only. Error bars shown are standard errors obtained from analysis of duplicate samples. The log–log plots were fit by linear regression. S.d.'s listed for the n* values in Table 1 were determined for each log–log plot by running linear regressions on a series of data sets, each missing one data point, to produce a set of slope values whose variance was then calculated. Thus, if there are a total of nine values in the full data set, there will be eight modified sets and eight resulting slope values whose s.d. from the mean is listed with the n* in the table.

### Data availability

Any additional data that support the findings of this study are available from the corresponding author upon request.

## Additional information

**How to cite this article:** Misra, P. *et al*. Rapid α-oligomer formation mediated by the Aβ C terminus initiates an amyloid assembly pathway. *Nat. Commun.* 7:12419 doi: 10.1038/ncomms12419 (2016).

## Supplementary Material

Supplementary Information Supplementary Figures 1-8

## Figures and Tables

**Table 1:** Amyloid nucleation and stabilization data.

**Peptides**	**Data Points**	**Slope**	***R***^**2**^ **of fit**	n*	***C***_**r**_ **(μM)**
K_2_Q_23_K_2_*	8	5.9	0.9875	3.9	2.9±0.5
K_2_Q_23_K_2_Aβ_30–42_	10	1.08	0.9067	−0.92±0.03	0.42±0.01
K_2_Q_23_K_2_Aβ_30–40_	7	2.86	0.9449	0.86±0.15	3.6±0.4
K_2_Q_23_Aβ_25–42_	7	1.88	0.9575	−0.12±0.02	0.08±0.01
HTT^NT^Q_23_K_2_†					≤0.1
HTT^NT^Q_23_P_10_K_2_†					0.28

^*^From ref. 8.

^†^From ref. 27.
