# Supplementary material for: Rapid α-oligomer formation mediated by the Aβ C terminus initiates an amyloid assembly pathway
Source: Nat Commun. 2016 Aug 22;7:12419. doi: 10.1038/ncomms12419 (PMC4996947; doi:10.1038/ncomms12419)
Supplement: Supplementary Information — Supplementary Figures 1-8 [file ncomms12419-s1.pdf]

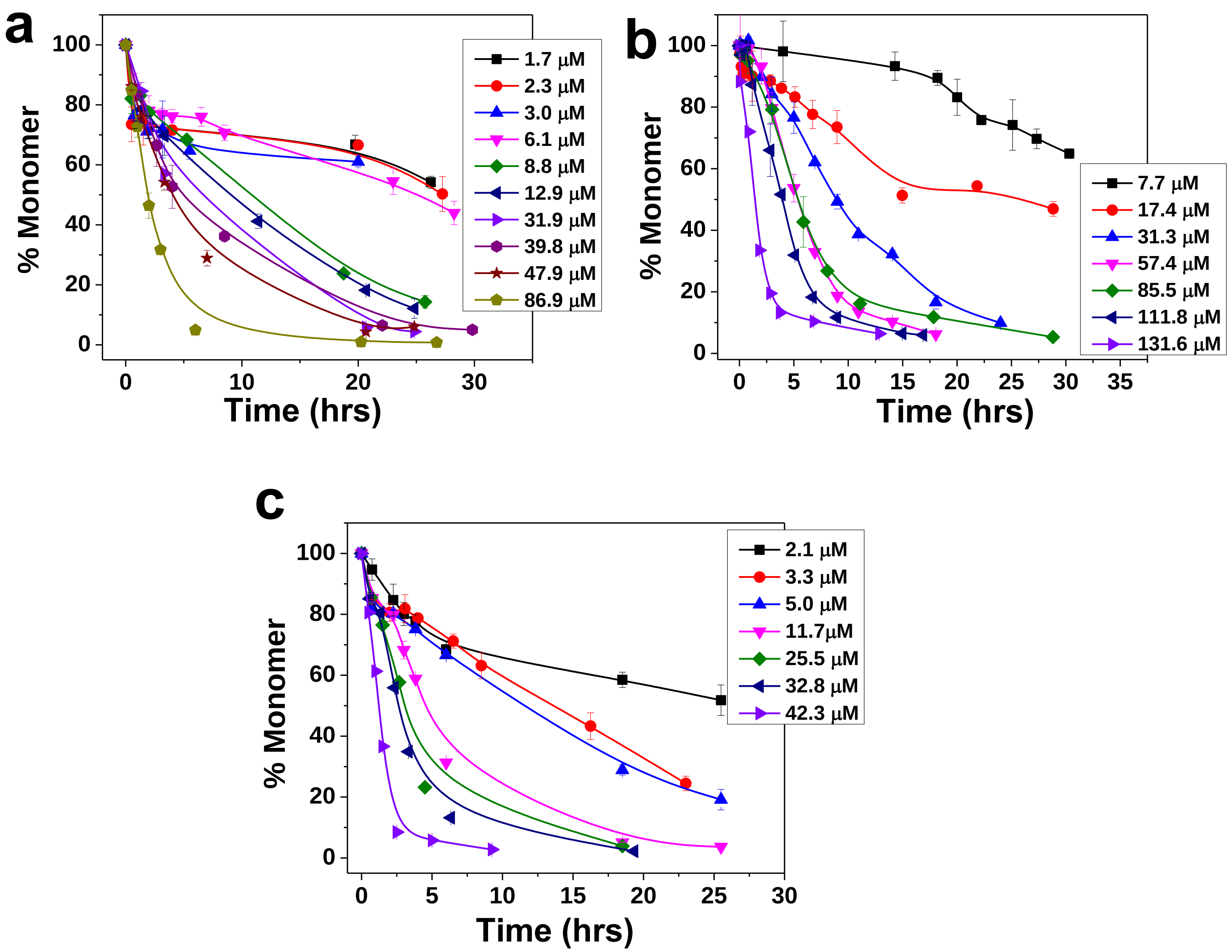


Supplementary Figure 1. Concentration dependent sedimentation assay data for the spontaneous aggregation of polyQ peptides used for nucleation kinetics analysis. Raw aggregation data for $K_2Q_{23}K_2A\beta_{30\text{-}42}$ (a), $K_2Q_{23}K_2A\beta_{30\text{-}40}$ (b), and $K_2Q_{23}A\beta_{25\text{-}42}$ (c).

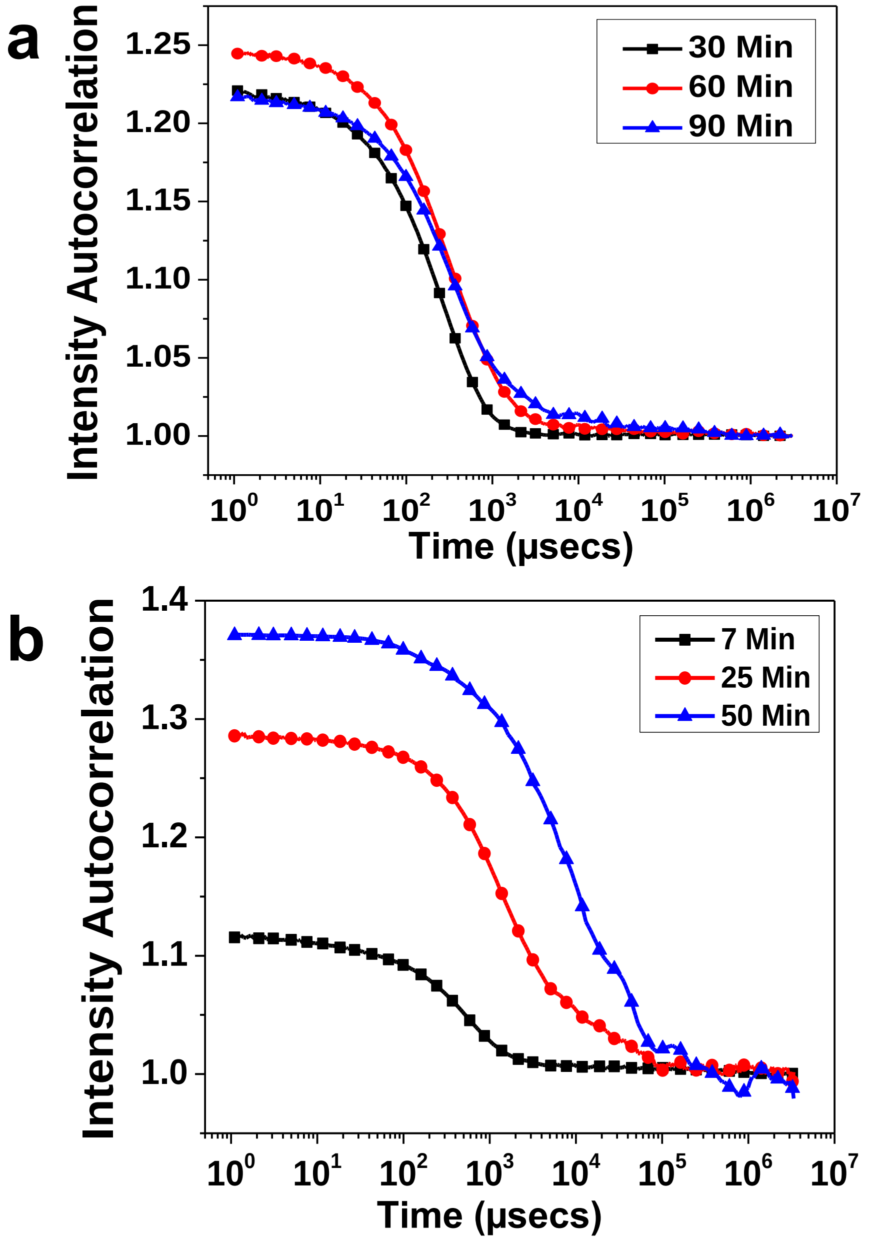


Supplementary Figure 2. Dynamic light scattering autocorrelations for aggregation time points in PBS at 37 °C for $K_2Q_{23}K_2A\beta_{30\text{-}42}$, 87 μM (a) and $K_2Q_{23}A\beta_{25\text{-}42}$, 25 μM (b).

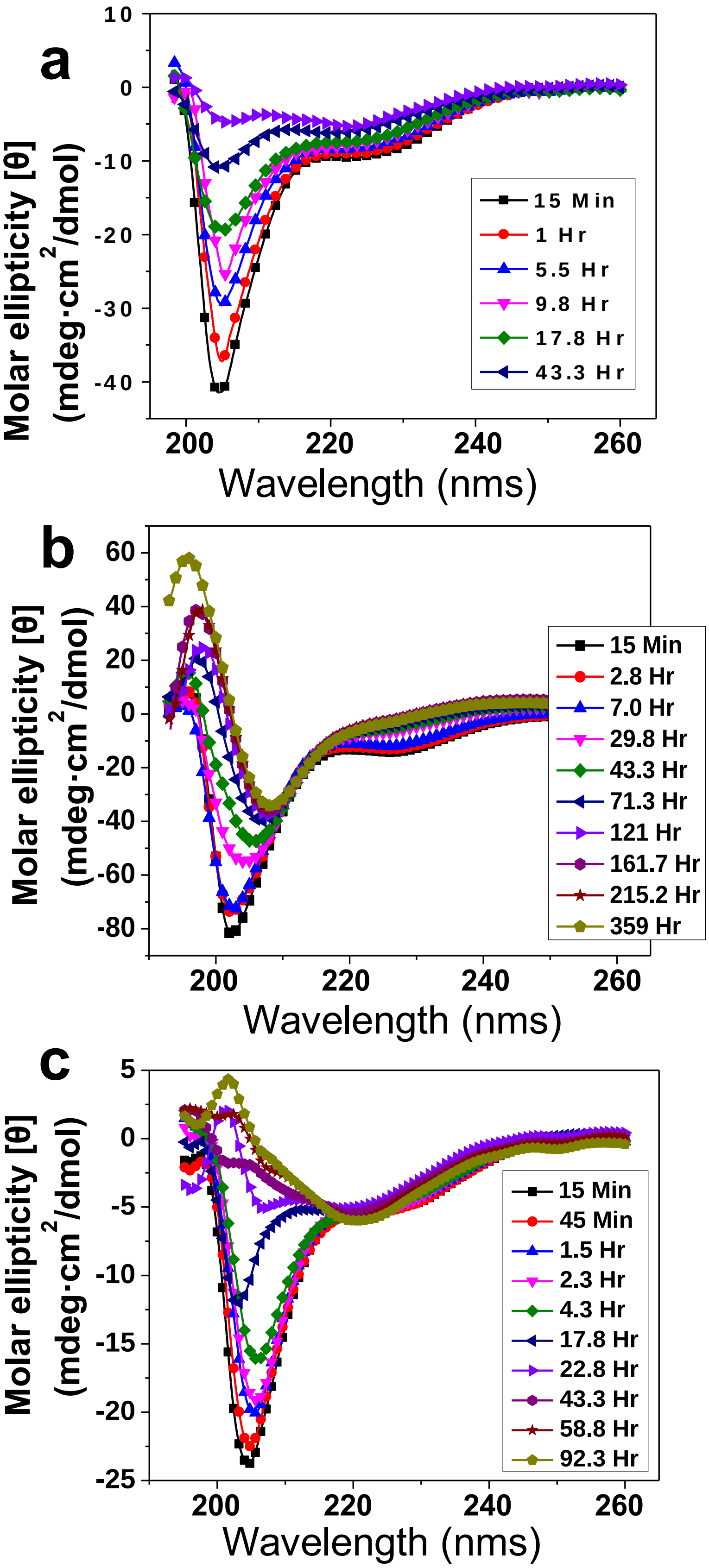


Supplementary Figure 3. Far UV circular dichroism spectra of time points of the aggregation at 37 °C in PBS of 6.1 μM $K_2Q_{23}K_2A\beta_{30-42}$ (a), 112 μM $K_2Q_{23}K_2A\beta_{30-40}$ (b), 3.4 μM $K_2Q_{23}A\beta_{25-42}$ (c).

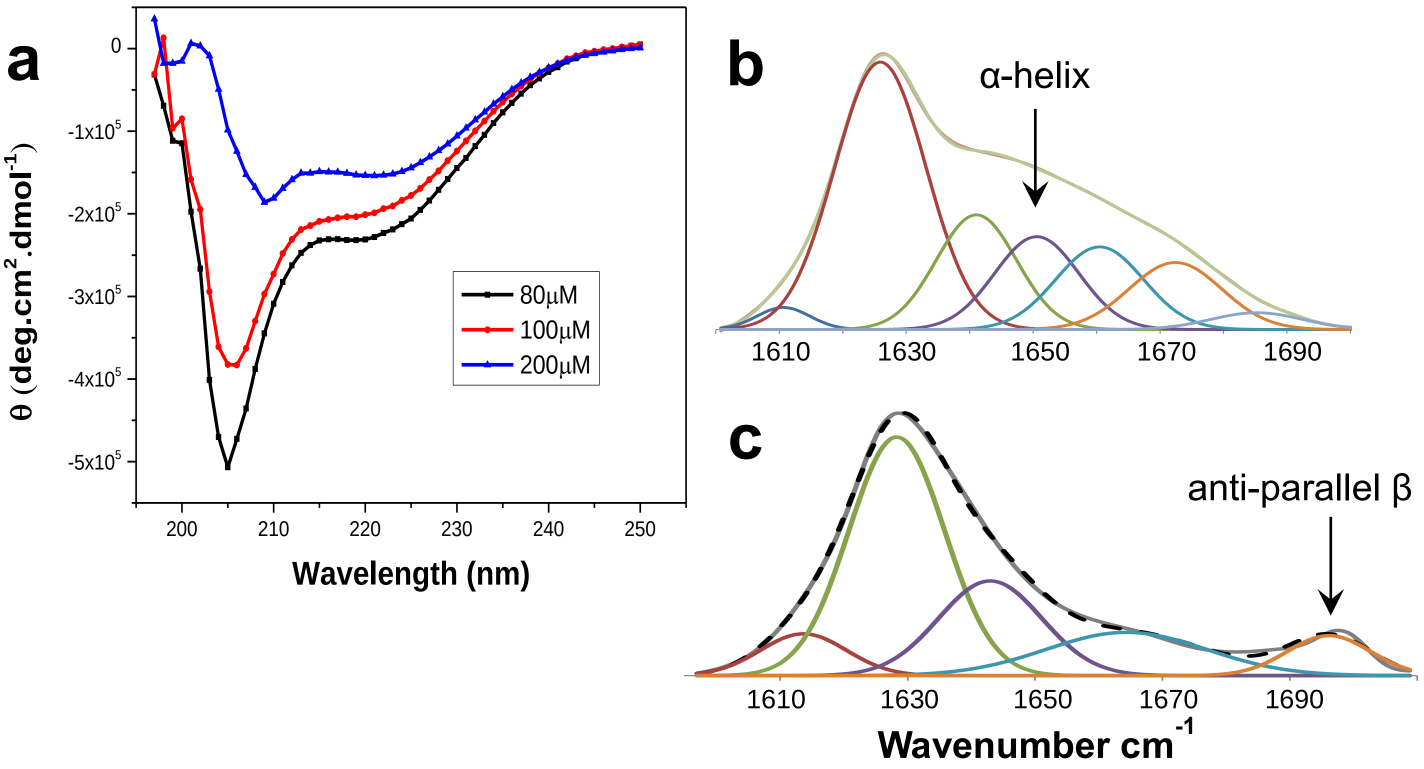


d

| Method | Conc (µM) | % α | % β | % parallel | % anti-parallel |
|---|---|---|---|---|---|
| CD | 80 | 34 | 0 | 0 | 0 |
| CD | 100 | 32 | 13 | 0 | 13 |
| CD | 200 | 21 | 30 | 3 | 27 |
| FTIR | 500 | 15 | 49 | - | - |

Supplementary Figure 4. Secondary structure analysis of A$\beta_{31\text{-}42}$ aggregates. a. Freshly disaggregated $K_4G_2A\beta_{31\text{-}42}$ was diluted in Tris.HCl, pH 7.4 to different concentrations and CD spectra collected immediately. b. Freshly disaggregated $K_4G_2A\beta_{31\text{-}42}$ was suspended in Tris.DCl, pH 7.4, $D_2O$ and the FTIR spectrum collected immediately. Note that the large β-sheet peak at ~1626 $cm^{-1}$ and the peak at ~1641 $cm^{-1}$ normally attributed to disordered chain are both shifted 2-3 $cm^{-1}$ compared to the peaks in panel c because the panel c spectrum was not obtained in $D_2O$. c. FTIR spectrum of mature A$\beta_{31\text{-}42}$ amyloid fibrils collected from an aggregation reaction in PBS, pH 7.4 showing a high frequency band often attributed to anti-parallel β-sheet. d. Total α- and β-contents from deconvolution of spectra in panels a and b. Total % β is the sum of the parallel and anti-parallel contributions to the CD spectra. Note that the secondary structures from the 500 µM FTIR spectrum continues the concentration dependent trends for 80-200 µM CD spectra. This suggests that in spite of light scattering by aggregates the CD deconvolution data are reasonably accurate. The trends suggest a model in which freshly dissolved peptide rapidly forms α-helical oligomers in a concentration dependent manner. However, the greater oligomer and monomer concentrations at higher peptide concentrations produce rapid nucleation of amyloid-like structures resulting in a diminution of α-structure and a corresponding increase in β-structure. Nonetheless, at 80 µM, a clean α-helix spectrum was collected.

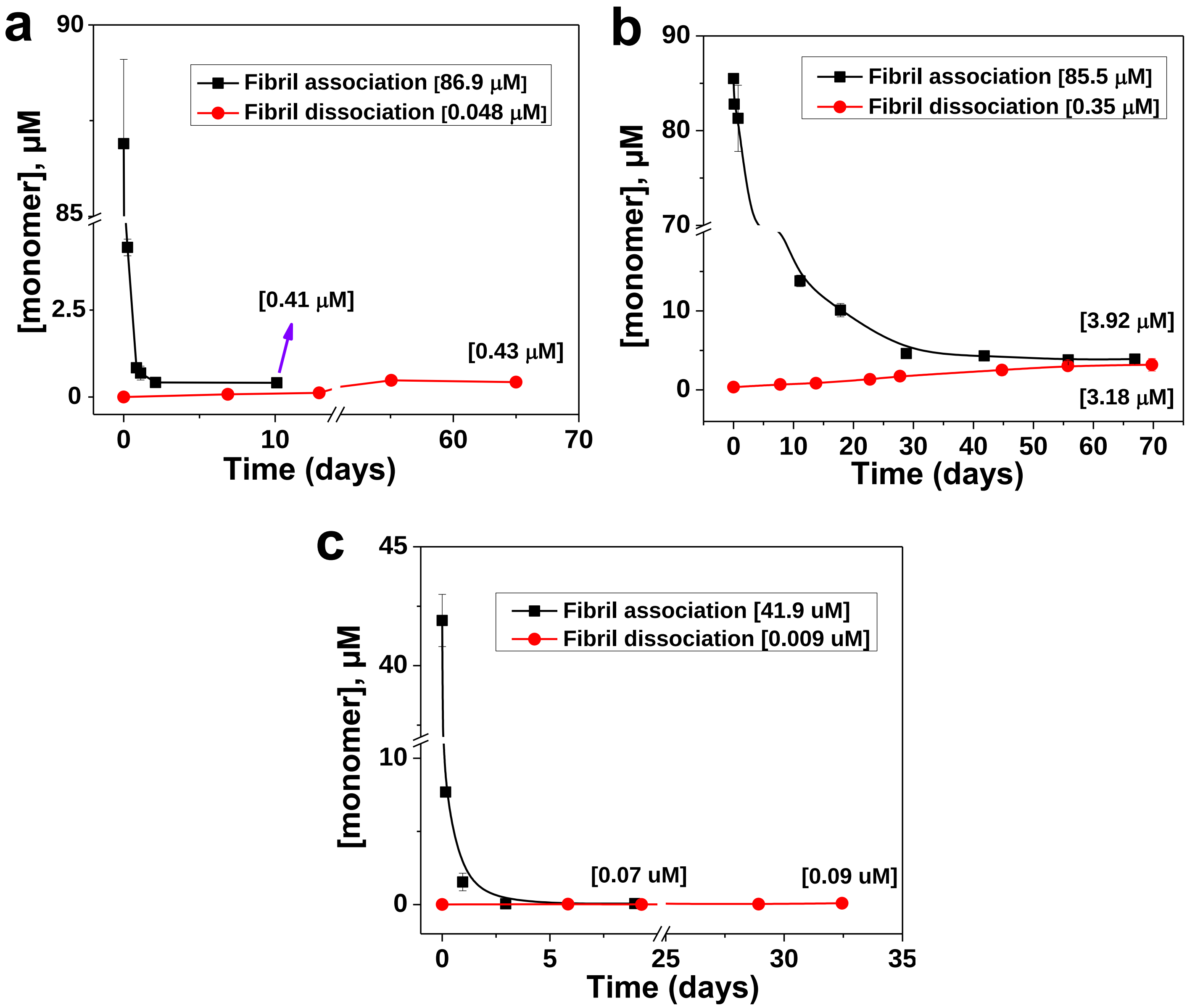


Supplementary Figure 5. Determination of equilibrium monomer concentrations ($C_r$) for polyQ-Aβ hybrid peptides from both the fibril association and fibril dissociation directions. a. $K_2Q_{23}K_2A\beta_{30\text{-}42}$; b. $K_2Q_{23}K_2A\beta_{30\text{-}40}$; c. $K_2Q_{23}A\beta_{25\text{-}42}$. Spontaneous amyloid formation reactions were initiated from freshly disaggregated monomer at the starting concentrations shown in the key for the fibril association (■) reaction and monitored until the last time point shown. At this point, a portion of the association reaction mixture was diluted approximately 10-fold into PBS to give the residual monomer concentrations shown in the key for the fibril dissociation (●) reaction. These diluted reactions mixtures, with total (i.e., monomer plus amyloid) peptide concentration well above the expected $C_r$ value, were incubated and monitored for the increase in monomer concentration as fibrils dissociate to restore equilibrium. The final monomer concentration in the association and dissociation reactions were averaged to generate the value shown in Table 1.

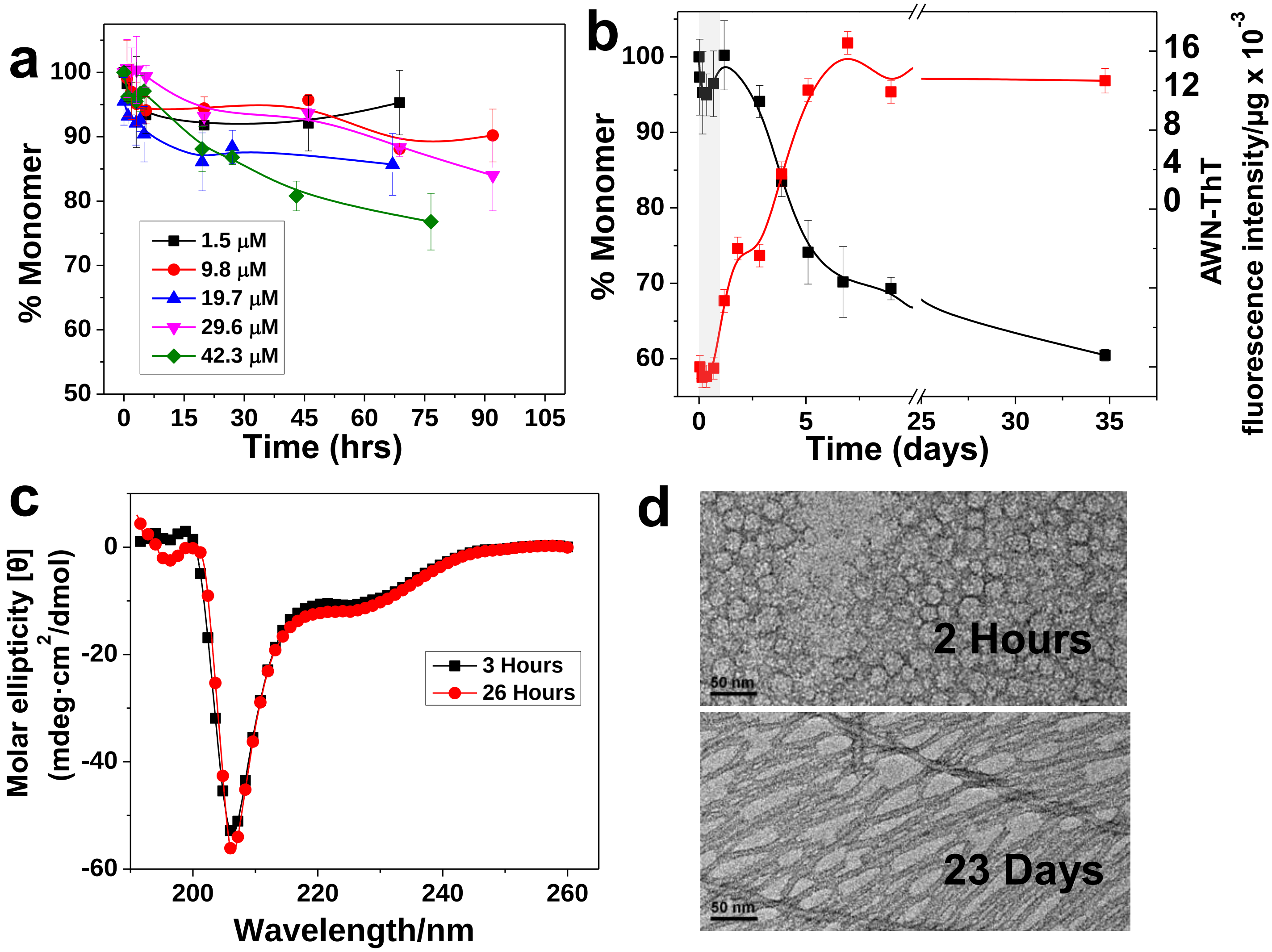


Supplementary Figure 6. Aggregation properties of $K_{10}G_2A\beta_{31-42}$ in PBS at 37 °C. a. Concentration dependence of aggregation assessed by HPLC sedimentation assay, showing that the peptide generates sedimentable aggregates over several days incubation when incubated at 10 µM or above. b. Aggregation at 22 µM assessed by sedimentation assay (■) and weight-normalized ThT assay (■), showing that for the first day of incubation (gray bar), the aggregates formed are ThT-negative and hence unlikely to possess β-structure. At later timepoints the aggregates exhibit progressively higher ThT intensities on a weight basis until 5-6 days, after which more monomers join aggregates, but the aggregates no longer change in their weight based ThT intensities. c. Circular dichroism spectra of the 22 µM incubation shown in panel b showing little change in the spectrum up to 24 hrs, during the period when only a few % of monomers have aggregated (panel a), and when the aggregates formed are non-β (panel d) Deconvolution of these curves (Methods) shows unchanging levels of secondary structure over this time period, with 22 % α-helix, 17 % β-sheet, 17% β-turns, and 44% unordered structure. d. Electron micrographs of time points of the 22 µM aggregation reaction showing a uniform distribution of spherical oligomers in the 25 nm range after two hours, corresponding to the non-β aggregates, and a uniform suspension of fibrils after 23 days incubation.

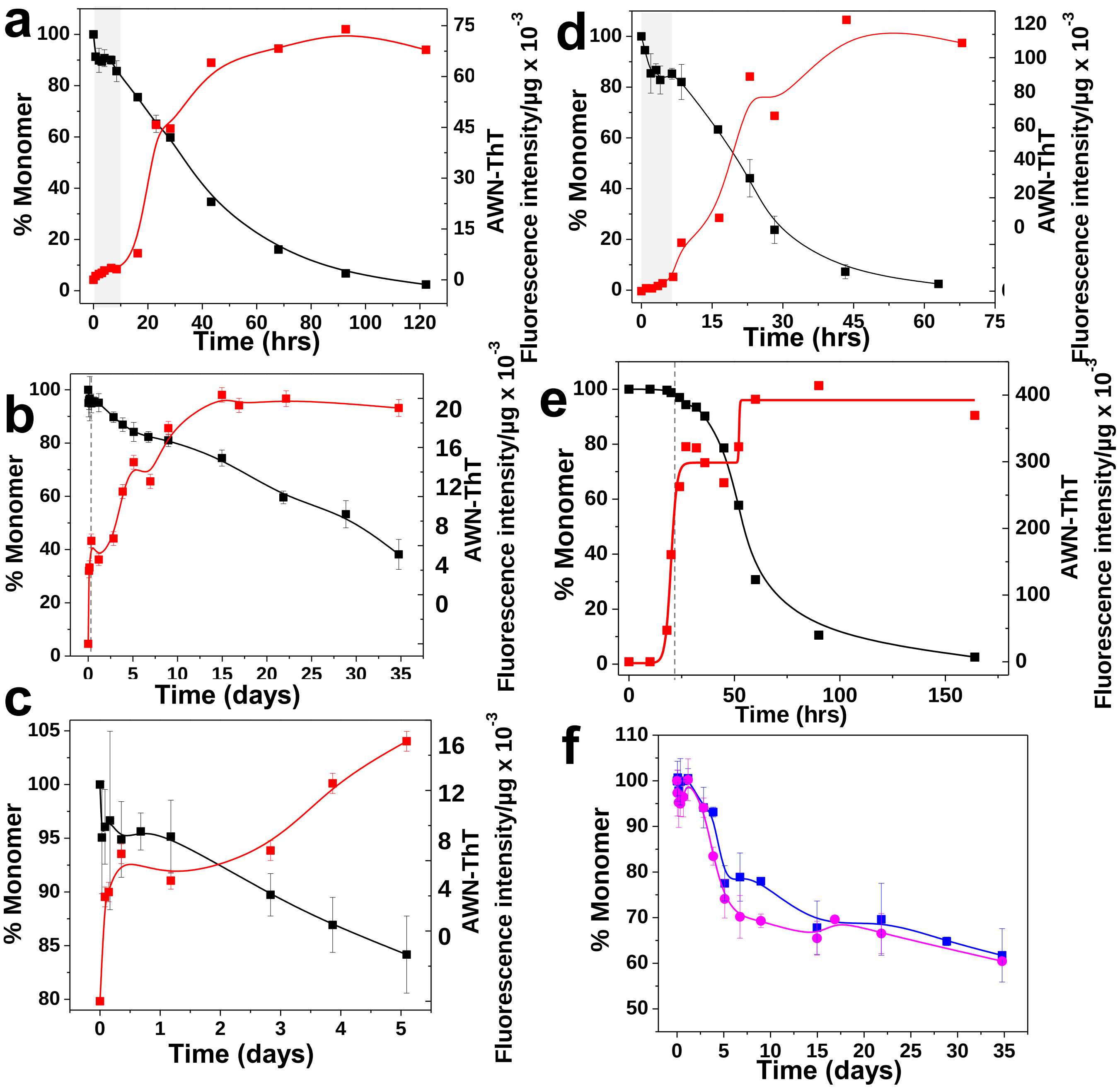


Supplementary Figure 7. Details of inhibition of polyQ-Aβ chimeric peptides by $K_{10}G_2Aβ_{31-42}$ in PBS at 37 °C. a – e. Weight normalized ThT fluorescence curves (■) plotted next to disappearance of monomeric chimeric peptide from solution (■; HPLC-sedimentation aggregation assay) for the following: a. $K_2Q_{23}K_2Aβ_{30-42}$; b,c. $K_2Q_{23}K_2Aβ_{30-40}$; d. $K_2Q_{23}Aβ_{25-42}$; e. $K_2Q_{23}K_2$; f. Time dependent aggregation of $K_{10}G_2Ab_{31-42}$ incubated alone (■) or in the presence of $K_2Q_{23}K_2Aβ_{30-40}$ (●) in the experiment described in Figure 6b. Shaded areas in panels a and d indicate early segments of the aggregation curve where sedimentable aggregates are detected while little or no ThT fluorescence is observed. In contrast the weight normalized ThT signal increases significantly at the earliest time points in b and e (dashed line), consistent with spontaneous aggregation of $K_2Q_{23}K_2$ and $K_2Q_{23}K_2Aβ_{30-40}$ occurring without any non-β aggregated intermediates but rather via direct formation of monomeric nuclei. a-d are the same reactions shown in Figure 6 a-c; e is for a starting concentration of 215 µM $K_2Q_{23}K_2$. Note that in panel c the % monomer scale is condensed but the ThT intensity scale is not. Note that while the sedimentation assay kinetics in panels a-d reflect only the polyQ-Aβ chimeric peptide (since it can be independently measured due to its unique HPLC elution position), the ThT data in panels a-d are for total ThT which may include signal from any amyloid fibrils of the inhibitor (Supplemental Fig. 5d ) that might be present.

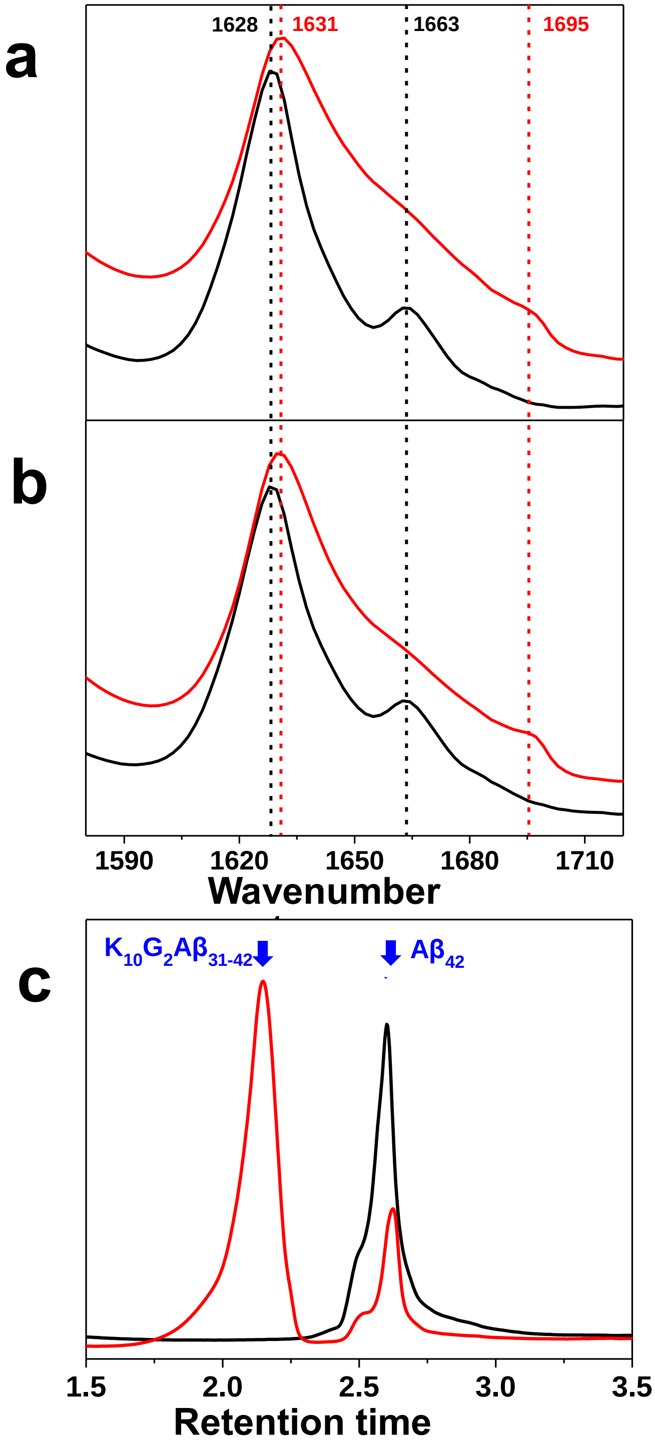


Supplementary Figure 8. Properties of $A\beta_{42}$ aggregates. a., b. FTIR spectra of aggregates isolated by centrifugation from spontaneous aggregation of $A\beta_{42}$ incubated alone (7.4 µM; black line) and in the presence of $K_{10}G_2A\beta_{31\text{-}42}$ (11.5 µM; red line) after reaction times of 4 hrs (a) or 24 hrs (b). The spectra change very little between 4 and 24 hrs. c. HPLC analysis of aggregates isolated by centrifugation from the $A\beta_{42}$ + $K_{10}G_2A\beta_{31\text{-}42}$ reaction (black trace) compared with a co-injection of a mixture of the two peptides (red trace). No inhibitor is detected in the aggregated product of the inhibition reaction. FTIR shows significant differences between the $A\beta_{42}$ aggregates formed in the presence and absence of inhibitor. The amyloid fibrils formed at 4 and 24 hrs from $A\beta_{42}$ alone (Fig. 7) exhibit a strong peak at 1663 $cm^{-1}$ that is normally assigned to turns. The aggregates formed at 4 and 24 hrs from $A\beta_{42}$ with inhibitor exhibit a peak at 1695 $cm^{-1}$ that is normally assigned to anti-parallel β-sheet. Both aggregates have strong β-sheet bands (1628 – 1631 $cm^{-1}$).
